# Biological nitrogen removal from low carbon wastewater

**DOI:** 10.3389/fmicb.2022.968812

**Published:** 2022-11-16

**Authors:** Kiprotich Kosgey, Phumza Vuyokazi Zungu, Faizal Bux, Sheena Kumari

**Affiliations:** Institute for Water and Wastewater Technology, Durban University of Technology, Durban, South Africa

**Keywords:** nitrogen removal, anammox process, DEAMOX process, bioelctrochemical process, autotrophic denitrification, low carbon wastewater

## Abstract

Nitrogen has traditionally been removed from wastewater by nitrification and denitrification processes, in which organic carbon has been used as an electron donor during denitrification. However, some wastewaters contain low concentrations of organic carbon, which may require external organic carbon supply, increasing treatment costs. As a result, processes such as partial nitrification/anammox (anaerobic ammonium oxidation) (PN/A), autotrophic denitrification, nitritation-denitritation and bioelectrochemical processes have been studied as possible alternatives, and are thus evaluated in this study based on process kinetics, applicability at large-scale and process configuration. Oxygen demand for nitritation-denitritation and PN/A is 25% and 60% lower than for nitrification/denitrification, respectively. In addition, PN/A process does not require organic carbon supply, while its supply for nitritation-denitritation is 40% less than for nitrification/denitrification. Both PN/A and nitritation-denitritation produce less sludge compared to nitrification/denitrification, which saves on sludge handling costs. Similarly, autotrophic denitrification generates less sludge compared to heterotrophic denitrification and could save on sludge handling costs. However, autotrophic denitrification driven by metallic ions, elemental sulfur (S) and its compounds could generate harmful chemicals. On the other hand, hydrogenotrophic denitrification can remove nitrogen completely without generation of harmful chemicals, but requires specialized equipment for generation and handling of hydrogen gas (H_2_), which complicates process configuration. Bioelectrochemical processes are limited by low kinetics and complicated process configuration. In sum, anammox-mediated processes represent the best alternative to nitrification/denitrification for nitrogen removal in low- and high-strength wastewaters.

## Introduction

Nitrogen in wastewater presents serious ecological challenges to the receiving water bodies, including eutrophication and toxicity to aquatic life. It is dissolved as ammonium (NH_4_^+^), nitrite (NO_2_^−^), nitrate (NO_3_^−^) and organic compounds (e.g., amino acids and CN^−^) in wastewaters (Wiesmann, 1994; Wang et al., 2012). When organics are degraded by microorganisms, organic nitrogen is transformed to NH_4_^+^ (Wiesmann, 1994). In mainstream wastewater, the concentration of NH_4_^+^ and organic nitrogen is about 40 and 20 mg-N/L, respectively (Wiesmann, 1994). Nitrogen and COD concentrations vary depending on the source of wastewater (Tables 1, 2). Chemical oxygen demand (COD) to nitrogen ratio (C/N) ratios in mainstream wastewaters are typically ≥2, while those in sidestream wastewaters generally contain lower concentrations (Li et al., 2018a). COD concentrations are high in industrial wastewaters (Table 2). For instance, effluents from a biodiesel plant, was reported to contain COD concentration of up to 403,540 mg/L (Mousazadeh et al., 2021).

**Table 1:** Nitrogen and COD concentrations in low- and high-strength wastewaters.

	NH_4_^+^ conc. (mg-N/L)	NO_3_^−^ conc. (mg-N/L)	COD conc. (mg/L)	C/N^a^	Remarks	References
1	1,000	-	810	0.8	Reject water	Hellinga et al. (1998)
2	223–229	-	1,090–1,270	5–6	Abattoir wastewater	Lemaire et al. (2008)
3	1,128 ± 141	0.77 ± 0.3	1956 ± 597	1–2.6	Reject water from digested sludge	Noutsopoulos et al. (2018)
4	66 ± 17	2.09 ± 0.2	4,244 ± 2,100	25.8–129.5	Reject water from primary sludge thickening unit	
5	142.7 ± 40.9	-	1,223 ± 336	4.8–15.3	Slaughterhouse wastewater	Dobbeleers et al. (2020)
6	1,500	-	2,700	1.8	Landfill leachate	Magrí et al. (2021)

a Calculated.

**Table 2:** Summary of nitrogen and COD concentrations in industrial and mining wastewaters.

	NH_4_^+^ conc. (mg-N/L)	NO_3_^−^ conc. (mg-N/L)	COD conc. (mg/L)	CN^−^/ SCN^−^ (mg/L)	C/N^a^	Remarks	References
1	-	-	-	840	-	Wastewater from gold leaching process	Song et al. (2021)
2	110–165	55-80	2,750	0.5–3.5/22–45	16–25	Coal gasification wastewater	Wang et al. (2012)
3	325	201	-	-	-	Wastewater from fertilizer factory	Leaković et al. (2000)
4	56–132	0.5-1.2	900–2000	-	7–36	Wastewater from petrochemical industry	Qin et al. (2007)

a Calculated.

**Figure 1:**
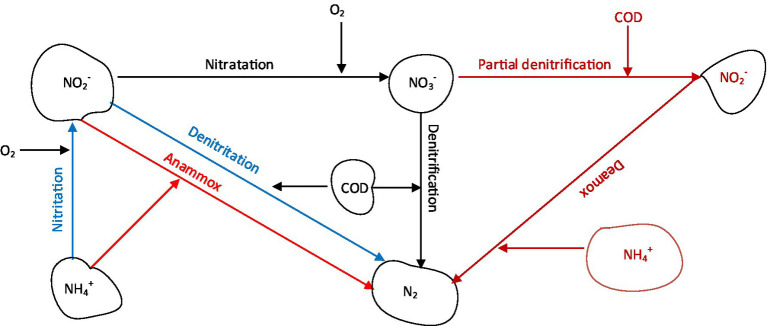
Schematic diagram of nitrogen removal processes.

Nitrification/denitrification represents the traditional nitrogen removal process that has been applied in the past several decades in municipalities across the globe (Grady et al., 2011). In this conventional process, NH_4_^+^ is sequentially oxidized to NO_3_^−^ by ammonia oxidizing bacteria (AOB) in the first stage, and by nitrite oxidizing bacteria (NOB) in the second stage, then the NO_3_^−^ is removed through denitrification in the last stage (Figure 1). However, the economic aspects of this process indicate that it is more costly compared to nitritation-denitritation and partial nitrification/anammox (anaerobic ammonium oxidation) processes because it consumes more COD, produces more sludge and requires more aeration (Hellinga et al., 1998; Kartal et al., 2013). These challenges relating to COD consumption are magnified in the treatment of low-COD wastewaters such as reject wastewater and landfill leachate, as supplementation with external COD sources is inevitable (Table 1).

Autotrophic denitrification could play a vital role in the treatment of wastewaters with high NO_3_^−^ concentrations such as some industrial wastewaters and groundwater (Pu et al., 2014). Compared to heterotrophic denitrification, this process is associated with lower sludge production and utilization of simple elements/ions, and could be applicable in treatment of wastewaters containing nitrogen and electron donors such as sulfide (S^2−^), SCN^−^ (thiocyanate), hydrogen gas (H_2_), etc. (Cardoso et al., 2006; Di Capua et al., 2019). However, autotrophic denitrification driven by metallic elements and ions as well as those driven by S and its compounds have been reported to generate harmful chemicals that require downstream treatment to avert environmental disasters (Di Capua et al., 2019).

The PN/A process has generated a lot of interest from water practitioners leading to the development of over 100 full-scale systems (Lackner et al., 2014a; Bowden et al., 2015). However, the process still requires further improvements to emerge as an efficient alternative for nitrogen removal. Moreover, there is need to develop the process for mainstream applications because of the associated benefits (Li et al., 2018a). Furthermore, PN/A converts a fraction of NH_4_^+^ to NO_3_^−^, which reduces process efficiency (Daverey et al., 2013), and would thus be necessary to incorporate systems for NO_3_^−^-removal downstream.

Cyanide (CN^−^) and its derivatives which are present in some industrial wastewaters such as coal gasification and gold/silver mining industries (Table 2), is highly toxic and its removal is necessary to avert environmental disasters (Kjeldsen, 1999; Chen et al., 2008). Some physical and chemical processes have been developed for its removal from wastewater, but these processes are generally characterized by high costs (Akcil et al., 2003). Therefore, biological processes have generated a lot of interest because they are cheap and safe, as they do not generate harmful secondary chemical wastes. Microalgae, plants, bacteria and fungi have all been determined to be able to remediate against CN^−^ contamination (Chapatwala et al., 1998; Ezzi and Lynch, 2002; Gurbuz et al., 2004). This study thus incorporates biodegradation of CN^−^ and its derivatives as they can be transformed to NH_4_^+^ and other nitrogen compounds (“Biological degradation of cyanide” and “Autotrophic denitrification”). Furthermore, since SCN^−^ can be utilized as electron donor (Pan et al., 2018), it is necessary to analyze their influence on nitrogen removal.

In the recent past, there have been reviews on different low carbon nitrogen removal processes, including those covering ANAMMOX-mediated processes (Adams et al., 2022), autotrophic denitrification (Di Capua et al., 2019; Li et al., 2022), and bioelectrochemical processes (Albina et al., 2019; Cecconet et al., 2020; Jung et al., 2020) among other biological processes (Winkler and Straka, 2019; Abeysiriwardana-Arachchige et al., 2020; Mohsenpour et al., 2021). Despite the useful insights presented in those previous reports, many aspects of low carbon nitrogen removal processes were not adequately addressed including the process kinetics, applicability at full-scale level, actual COD consumption/g-N removed, by-product and sludge generation. Furthermore, the previous reviews either focused on aspects of a single technology without the inclusion of other low-carbon processes in the discussion, or they did not critically review these technologies with respect to influencing factors. Therefore, these subjects are discussed in this review with reference to experimental data collected from different systems (laboratory-, pilot- or full-scale). The choice of the nitrogen removal processes to be included in this review was informed by process readiness for application at pilot-scale and full-scale installations.

## Nitritation-denitritation

Theoretically, 2.86 g of COD would be required to remove 1 g NO_3_^−^-N (Hellinga et al., 1999; Daigger, 2014). However, considering that substantial amount of COD would be oxidized during aeration phase, the actual required C/N ratios for complete nitrogen removal is higher (5–7; (Hellinga et al., 1999; Sahinkaya et al., 2014). On the contrary, 1.94–3\u00B0C/N ratios have been reported to be sufficient for nitrogen removal through the NO_2_^−^ route (nitritation-denitritation) (Hellinga et al., 1999; Van Kempen et al., 2001; Bernat et al., 2016; Table 3). This nitrogen removal option saves approximately 40% COD and 25% aeration costs compared to nitrification/denitrification since NH_4_^+^ is only oxidized to NO_2_^−^ as opposed to NO_3_^−^ in the case of nitrification/denitrification (Hellinga et al., 1999; Van Kempen et al., 2001; Noutsopoulos et al., 2018; Dobbeleers et al., 2020).

**Table 3:** Nitrogen removal in laboratory-, pilot- and full-scale nitritation-denitritation systems.

	Influent NH_4_^+^ conc. (mg-N/L)	C/N ratio	Operational temperature (°C)	HRT (days)	NH_4_^+^ conversion rates (%)	NRR (kg-N/m^3^-day)	NRE (%)	Remarks	References
1	4,559 ± 201	-	36 ± 1	-	60	-	-	250 l system	Ganigué et al. (2010)
2	>1,000	2.4	30–40		>90	-	65-95^a^	Full-scale system	Mulder et al. (2001)
3	1,135	2.2	30–32	2.3	80^b^	1.0	67	5.65 l CSTR	Fux et al. (2006)
4	1,216	1.94		1.0–1.6	95^c^	3.0–3.6	93	6 l SBR with continuous sludge addition	
5	400–700	-	35	-	1-3^d^	-	95-98	7 l CSTR operated with sequencing of operations	Claros et al. (2012)
6	1750-1900^b^	-	-	1.86-2.71/2.71	79–98	0.63	95	Two-stage SBRs (10 l/6 l)	Zhang et al. (2019)
7	200 ± 18^e^	3	20–22	0.71–3.33	-	-	100	5 l SBR	Bernat et al. (2016)

a From Figure 6 in the source file.

b Estimated in Figure 2 in the source file.

c Estimated from Figure 3 in the source file.

d Figure 3a in the source file;

e NO_2_^−^.

Coupling nitritation with denitritation requires parameter control for successful implementation, including the control of pH, DO, temperature and SRT/HRT. This is done in order to suppress the growth of NOB while promoting the growth of AOB. Generally, temperature (30°C–40°C), pH (7–8), DO (<0.5 mg/L) and HRT/SRT (~1 day) have been reported to suppress NOB growth (Van Kempen et al., 2001). This process, referred to as SHARON (single reactor system for high activity ammonia removal over nitrite), is operated without sludge retention using concentrated wastewaters such as landfill leachate and reject wastewater from anaerobic digestion (Van Kempen et al., 2001; Vilar et al., 2010).

At 5-20°C, NO_2_^−^ oxidizers grow faster than NH_4_^+^ oxidizers, leading to NO_3_^−^ generation (Hellinga et al., 1998). However, at higher temperatures (30°C–40°C), the reverse is true. This difference in growth rates informs the design of SHARON systems that seek to limit nitratation while favoring nitritation. In the process, a combination of elevated temperatures (30-40°C) and short HRTs (~1 day) lead to NOB washout (Hellinga et al., 1998; Fux et al., 2006). In essence, the high growth rates of AOB compensate for the biomass washed out from the system. Furthermore, the high temperatures and high concentrations of NH_4_^+^ and NO_2_^−^ also limit the activities of NOB as they dissociate to FA (free ammonia) and FNA (free nitrous acid), respectively, both of which are more toxic to NOB compared to AOB (Anthonisen et al., 1976; Duan et al., 2019). Indeed, inhibitory FA and FNA concentrations for NOB are 0.10–1.00 mg/L and 0.011–0.070 mg/L, while that of AOB is 10.00–150.00 mg/L and *ca.* 0.40, respectively (Anthonisen et al., 1976; Chen et al., 2020).

For complete nitrogen removal from wastewater using nitritation-denitritation, supplementation of COD is necessary for low COD wastewaters (Figure 1). The process could be implemented in a continuously stirred system in which a COD source is added during an unaerated phase (Lemaire et al., 2008). Since aeration causes pH to drop and denitritation causes pH to rise, the pH could be used to control aeration and dosing of COD sources. This strategy is beneficial as the pH is adjusted during nitritation and denitritation, eliminating the need for addition of alkali (Hellinga et al., 1998). If the influent wastewater contain high C/N ratios (>2), the same wastewater could be utilized as the COD source by implementing a step-feeding strategy as was demonstrated by Lemaire et al. (2008). Other methods for process control include sequencing of operations in order to set time intervals. ORP (oxidation–reduction potential)-based strategies could also be used to regulate the process, as demonstrated previously (Claros et al., 2012).

Lai et al. (2004) reported 96%–98% removal of influent NH_4_^+^ in a 6.5 m^3^ nitritation-denitritation pilot-scale SBR system which was fed with 1.6–1.9 kg COD-equivalent ethanol per kg N removed. The low HRT (0.88 days), and high temperatures (35 ± 2°C) enhanced NOB suppression in line with Hellinga et al. (1998). However, the reported C/N ratios were lower than those reported by (Van Kempen et al., 2001), possibly because of the sequencing of reactor operations in the SBR leading to lower COD consumption by aerobic heterotrophic bacteria compared to continuous dosing in the CSTR. Similar findings were reported in a comparative study of nitritation-denitritation by Fux et al. (2006) in a CSTR and SBR, in which better nitrogen removal in SBR compared to CSTR was observed (Table 3). In addition, lower HRTs were achieved (1–1.6 days) in SBR mode compared to CSTR mode of operation (2.3 days). As a result, operating reactors in CSTR mode would require the application of larger reactors in order to achieve the same performance as SBRs (Fux et al., 2006). Notwithstanding this finding, CSTRs have been applied in full-scale SHARON systems for nitrogen removal (Mulder et al., 2001). Therefore, comparative studies of full-scale nitritation-denitritation systems based on COD consumption/nitrogen removal could be necessary for a better understanding of the impact of reactor configuration.

Autotrophic denitritation could be advantageous to heterotrophic denitritation due to less sludge production (“Autotrophic denitrification”). Co-occurrence of NH_4_^+^ and inorganic electron donors in some industrial wastewaters (Table 2) presents an opportunity for nitrogen removal through autotrophic denitritation. Indeed, Qian et al. (2016) demonstrated the feasibility of nitritation-denitritation in an autotrophic system. However, low process kinetics and production of SO_4_^2−^ and other chemicals (“Autotrophic denitrification”) could limit the application of autotrophic denitrification.

## ANAMMOX-mediated nitrogen removal

ANAMMOX process has mainly been applied to treat high-strength NH_4_^+^-containing wastewater (Lackner et al., 2014a). This include reject wastewater, landfill leachate and industrial wastewater (Azari et al., 2017; Yao et al., 2022). Meanwhile, research on the development of mainstream anammox is ongoing. In general, the design of a treatment process depends on a number of variables, including the characteristics of the influent, the concentration of active biomass, and the flow rates. In PN/A systems, the functions of AOB and anammox bacteria (AMX) are coupled together (“Partial nitritation-ANAMMOX”), while in DEAMOX systems, the activities of nitrate-reducing denitrifying microorganisms are coupled with AMX (“Denitrifying ammonium oxidation”).

### Partial nitritation-ANAMMOX

In PN/A systems, AOB and AMX sequentially work together to oxidize NH_4_^+^ to nitrogen gas (N_2_) without the consumption of COD (Equations 1–3; (Daverey et al., 2013). However, during this process, approximately 11% of the influent NH_4_^+^ is converted to NO_3_^−^ (Equation 3), which can be removed downstream of the treatment process by mixing with COD containing wastewater if the effluent limits are exceeded (Mulder et al., 2012), otherwise PN/A alone has been reported to be adequate in many plants (Lackner et al., 2014a). In case of NO_3_^−^ and NH_4_^+^ presence in the influent such as in, industrial wastewaters (Table 2), activities of AMX could be coupled with that of partially denitrifying microorganisms that could generate NO_2_^−^ for the AMX in a process called DEAMOX (DEnitrifying AMmonium OXidation; “Denitrifying ammonium oxidation”; Le et al., 2019a,b).


(1) $${\mathrm{NH}}_{3}+1.5O_{2}\to {\mathrm{NO}}_{2}^{-}+H_{2}O+H^{+}$$

(2) $${\mathrm{NH}}_{3}+1.3{\mathrm{NO}}_{2}^{-}+H^{+}\to 1.02N_{2}+0.26{\mathrm{NO}}_{3}^{-}+2H_{2}O$$

(3) $${\mathrm{NH}}_{3}+0.85O_{2}+H^{+}\to 0.44N_{2}+0.11{\mathrm{NO}}_{3}^{-}+1.43H_{2}O+0.14H^{+}$$

As of 2014, over 100 full-scale PN/A systems had been developed, majority of which were based on SBR configuration (Lackner et al., 2014a). However, despite the progress, this excellent technology is still facing numerous challenges including occasional plant failures, foaming, biomass washout, NOB/heterotrophic bacterial competition for oxygen with AOB and NO_2_^−^ with AMX, etc. (Lackner et al., 2014a). Therefore, further improvements of this technology is imperative for sustainable and efficient nitrogen removal.

The NOB and aerobic heterotrophic bacteria could compete for oxygen with AOB in PN/A systems. In addition, NOB and denitrifying heterotrophic bacteria compete for NO_2_^−^ with AMX (Li et al., 2018a). Successful implementation of PN/A systems thus requires strict control of conditions in order to limit the growth of organisms competing with AOB and AMX. Heterotrophic bacterial growth is generally limited through regulation of influent C/N ratios to ≤1 (Li et al., 2018a,b). As a result, sidestream (high-strength: >100-mg-N/L) wastewater is suitable for treatment in PN/A system unlike mainstream wastewaters which are associated with high C/N ratios (Li et al., 2018a).

Selective retention of biomass rich in the desired microorganisms (biofilms) could also be implemented, while that rich in undesired microorganisms (flocs) is wasted (Park et al., 2015). This could be achieved by limiting HRT and SRT in the respective reactors to certain set limits that would allow the growth of the desired microorganisms (AOB/AMX; Han et al., 2016). However, implementation of this strategy varies depending on the reactor configuration. For instance, SRT in SHARON reactors which are usually of CSTRs configuration is basically equal to HRT, while in carrier-based systems, SRTs are indefinite and biomass detachment from the carriers could only be induced through increased shear forces (Syron and Casey, 2008). It is noteworthy that although inducing biomass detachment from the carriers could achieve the intended aim of removing the biomass, the rate of detachment is challenging to control and could lead to unprecedented influence on process performance.

Among the developed reactor configurations, SBR is the most popular because of its simplicity and flexibility (Lackner et al., 2014a). However, the NRRs of this configuration is lower than that of continuous systems such as MBBRs (moving bed biofilm reactors; Lackner et al., 2014a; Table 4). Other systems such as MABRs (membrane aerated biofilm reactors) are complex in nature as the membrane lumens need to be pressurized with air which then diffuse into the bulk liquid, and MBRs (membrane bioreactors) are associated with high energy costs and fouling (Reij et al., 1995; Van Der Star et al., 2008).

**Table 4:** Nitrogen removal in full-scale PN/A systems.

	Influent NH_4_^+^ conc. (mg-N/L)	Influent NO_2_^−^/NH_4_^+^	NRR (kg-N/m^3^-day)	NRE (%)	Effluent NO_3_^−^/NH_4_^+^ (%)	Remarks	References
1	1,000-2700^a^	1.4 ± 0.6	0.32	86 ± 9	5	Two-stage system consisting of SBRs	Magrí et al. (2021)
2	750-1500^b^	1.31	7.1	-	25	Two-stage SHARON-ANAMMOX	van der Star et al. (2007)
3	960 ± 110	Single stage PN/A	0.18	72-85^c^	8-34	550 m^3^ SBR	Lackner et al. (2014b)
4	890 ± 89		-	>85	5-13^d^	393 m^3^ MBBR	Klaus et al. (2017)
5	1,043		0.63	90	2–8	350 m^3^	Christensson et al. (2013)
6	855		1.2	88	8–22	50×4 m^3^	
7	690	1.7-2^d^	3.0	80	3^d^	415 m^3^ 5-stage system with gel-encapsulated biomass	Isaka et al. (2017)
8	580 ± 90	Single stage	1.8^e^	>80	~12^f^	256 m^3^ full-scale PN/A MBBR	Dimitrova et al. (2020)

a Feed to nitritation reactor.

b Estimated from Figure 4 in the source file.

c Ammonium removal.

d Estimated from Figure 7 in the source file.

e g-N/m^2^-day.

f Estimated from Figure 2 in the source file.

Community analyses of ANAMMOX-mediated systems have shown the co-existence of bacteria, viruses, archaea and protozoans in these systems (Suarez et al., 2015). The relationship between these microorganisms is complex, and many things are not yet clearly understood. Some organisms complement each other in terms of sub-division of metabolic activities and generation of essential compounds for growth, while others are predatory in nature (Suarez et al., 2015; Lawson et al., 2017). In ANAMMOX-mediated systems, the presence of all these microbes is dependent on many factors, such as the operating conditions and mode of biomass growth, and as a result could change over time (Park et al., 2015; Suarez et al., 2015).

Within the AMX community, dominance of *Candidatus Brocadia* spp. has been associated with the presence of organic carbon and high substrate concentrations because of their high growth rates and mixotrophic lifestyles (Park et al., 2017a). On the other hand, *Candidatus Kuenenia* spp. have been suggested to dominate in systems with limited substrate concentrations because of their higher affinity for NO_2_^−^ (*K*-strategists) compared to the other species within the AMX community (Van Der Star et al., 2008). The factors influencing the growth dynamics of other bacteria within the AMX community such as *Candidatus Jettenia* spp. and *Candidatus Anammoxoglobus* spp. are yet to be established. *Candidatus* Scalindua spp. are marine in nature (Wei et al., 2016), but its detection in PN/A systems with low salinity as well as in soil has been reported (Wang and Gu, 2013).

Operating conditions have also been reported to influence the growth of AOB and NOB within the ANAMMOX-mediated systems. The growth of AOB-affiliated *Nitrosomonas* spp. which are regarded as *r*-strategists because of their high growth rates have been associated with high substrate concentrations, while *Nitrosospira* spp. which are regarded as *K*-strategists because of their high affinity for substrate have been associated with low substrate concentrations (Awolusi et al., 2015). The factors driving the growth of other AOB-affiliated bacteria such as *Nitrosococcus* spp., *Nitrosovibrio* spp. and *Candidatus Nitrosoglobus* spp. is not well elucidated in the currently available literature and could be investigated in future studies. On the other hand, *Nitrospira* spp. are regarded as *r*-strategists within the NOB community, while *Nitrobacter* spp. are regarded as *K*-strategists. *Nitrolancea hollandica*, which also belong to NOB community, have been detected in ANAMMOX-mediated systems but their dominance has not been reported (Gu et al., 2018).

### Denitrifying ammonium oxidation

Treatment of NO_3_^−^-containing wastewater theoretically requires 2.86 g-COD/g-N for complete denitrification (Daigger, 2014). Systems receiving wastewaters with low COD would thus require supplementation of COD from external sources. This would increase the cost of treatment and complicate the treatment process. Therefore, partial denitrification (PD) which consumes less theoretical COD (1.14 g-COD/g-N), could be economically viable when combined with other processes such as ANAMMOX (DEAMOX; Cao and Zhou, 2019).

The success of DEAMOX process, however, relies on the success of PD. The PD process, in turn, depends on many factors including pH, NO_3_^−^ concentration, active microorganisms, nature of carbon sources, etc. (Glass and Silverstein, 1998; Xie et al., 2017; Le et al., 2019a,b). In some studies, it has been reported that the pH influences the accumulation of NO_2_^−^ (Cao and Zhou, 2019). For instance, at pH values of 7.5, 8.5 and 9.0, NO_2_^−^ concentrations of 250, 500 and 900 mg-N/L, respectively, were reported in a denitrification system fed with 2,700 mg-N/L (Glass and Silverstein, 1998). In some other studies, it has been suggested that C/N ratios influence the accumulation of NO_2_^−^ (Ma et al., 2020), while in others, contrary findings relating to C/N ratios have been made (Le et al., 2019a). According to Le et al. (2019a) and Le et al. (2019b), C/N ratios have no influence on the efficiencies of PD, which is in agreement with Sijbesma et al. (1996).

Some microorganisms can only partially reduce NO_3_^−^ to NO_2_^−^, while others can reduce both compounds to N_2_, and others can only reduce NO_2_^−^ to N_2_ (Xie et al., 2017). Therefore, it is possible to manipulate the operating conditions in order to enhance the growth of the required microorganisms that contribute to accumulation of NO_2_^−^ (Table 5). In addition, it could also entail the supply of a suitable carbon source preferred by the microorganisms of interest.

**Table 5:** Nitrogen removal in laboratory- and pilot-scale DEAMOX systems.

	Influent NH_4_^+^ conc. (mg-N/L)	Influent NO_3_^−^ conc. (mg-N/L)	PD eff. (%)	NRR (kg-N/m^3^-day)	NRE (%)	C/N ratio	Key microorganisms	Remarks	References
1	60	-	-	-	80 ± 4	2.6	Biofilm: *Ca. Jettenia* spp. (17.83%), *Ca. Kuenenia* spp. (2.62%) and *Thauera* (5.27%)	Two-stage denitratation/ANAMMOX-nitrification system	Ma et al. (2017)
2	500	530^a^	-	0.98	99.9	-	*Ca. Brocadia* spp. (57%), *Ca. Kuenenia* spp. (43%)	Abundance at family level: ANMED-2D (39%); uc_*Phycisphaerales* (15%); *Rhodocyclaceae* (10%); *Fimbriimonadaceae* (7%); *Brocadiaceae* (5%); uc_*envOPS12* (4%); *Methylomirabiliaceae* (3%)	Xie et al. (2017)
3	30–43	107-137	-	-	>90	2.02^b^	*Ca. Brocadia* spp. (~68%)^c^; *Ca. Jettenia* spp. (~0.5%)^c^; *Denitratisoma* (2.3–2.7%); *Thauera* (4.8–5.4%).	Two-stage PdN-ANAMMOX (5 l SBR coupled with 3.2 l up-flow anaerobic sludge blanket reactor)	Du et al. (2019)
4	50	50	96	-	82–93	2.6	*Ca. Brocadia* spp. (0.33%); *Ca. Kuenenia* spp. (0.23%); *Thauera* (61.53%);	Acetate-fed laboratory-scale 6 l system	Du et al. (2017)
5	50	50	67-87	-	85–90	3	*Ca. Kuenenia* spp. (1.01%); *Thauera* (45.17%); *Denitratisoma* (0.65%);	Ethanol-fed laboratory-scale 6 l SBR	
6	50–78	51–104	-	-	41–82	2–4	*Thauera* (43.6%-biofilm and 57.5%-suspension); *Ca. Brocadia* spp. (1.6%-biofilm and 0.1%-suspension)	Sodium acetate-fed laboratory-scale system	Zhang et al. (2022)
7	31–33	-	38	1.2 ± 0.7^d^	>77	2-3^e^	-	Fermentate-fed	Ladipo-Obasa et al. (2022)
8	0	40	64.4 ± 14.5	0.11–0.13	-	4.3–6.1^f^	*Comamonadaceae* (39.2%), *Hyphomicrobiaceae* (14.7%), *Flavobacterium* (12.3%), *Ignavibacteriaceae* (11.1%)	Hydrogenotrophic PD MBBR	Kamei et al. (2022)
9	29±4^g^	-	80-97	-	22-75^h^	2.2-13	*-*	360 l multi-stage system	Le et al. (2019b)

a NO_2_^−^.

b Calculated based on data in Table 2 in the source file.

c Calculated from Figure 4b in the source file.

d g-N/m^2^-d.

e Influent COD excluding the added COD.

f H2/N ratio.

g Total inorganic nitrogen.

h Estimated from Table 1 in the source file.

Accumulation of NO_2_^−^ (PD) by *Paracoccus denitrificans* was reported by Blaszczyk (1993) in a system fed with a media containing NO_3_^−^ and different carbon sources. However, the amount of NO_2_^−^ accumulation varied with the carbon sources, in agreement with Du et al. (2017) who reported higher efficiencies of PD with acetate compared to ethanol. In addition, Blaszczyk (1993) reported that the NO_2_^−^ was consumed once the NO_3_^−^ was depleted, possibly because: (i) slow induction of NO_2_^−^ reductase in comparison to NO_3_^−^ reductase; (ii) inhibition of NO reductase by NO_3_^−^; (iii) unbalanced reduction of NO_3_^−^ and NO_2_^−^; and (iv) inhibition of NO_2_^−^ reductase by NO_3_^−^. On the other hand, Martienssen and Schöps (1999) reported that species such as *Staphylococcus* could only reduce NO_3_^−^ to NO_2_^−^, while others such as *Bacillus niacini* could reduce NO_3_^−^ with transient NO_2_^−^ accumulation, and *Pseudomonas pseudoalcaligenes* could reduce NO_3_^−^ to N_2_ without NO_2_^−^ accumulation. Denitrifying anaerobic methane oxidizing (DAMO) archaea within the family of *ANME-2D* have also been reported to possess ability to reduce NO_3_^−^ to NO_2_^−^ (Equation 4; Xie et al., 2017). Therefore, with proper selection of carbon source and strains of desired microorganisms, it is possible to treat wastewaters such as industrial wastewaters rich in both NO_3_^−^ and NH_4_^+^ (Table 5) by coupling ANAMMOX with denitrification (DEAMOX). Indeed, Xie et al. (2017) reported complete removal of NO_2_^−^ and NH_4_^+^ in a MABR whose membrane lumens were pressurized with methane. In their study, following metagenomic analyses, it was concluded that DAMO archaea within *ANME-2D* family used methane as carbon source to reduce the NO_3_^−^ produced by AMX to NO_2_^−^, which was subsequently re-utilized in the ANAMMOX process. Xie et al. (2017) estimated that DAMO bacteria removed 10% of the nitrogen fed into the system, while DAMO archaea and AMX bacteria removed the remainder (90%). Within the ANAMMOX community, *Ca. Brocadia* spp. and *Ca. Kuenenia* spp. were detected at 57 and 43%, respectively (Table 5).


(4) $${\mathrm{CH}}_{4}+4{\mathrm{NO}}_{3}^{-}\to 4{\mathrm{NO}}_{2}^{-}+2H_{2}O+{\mathrm{CO}}_{2}$$

Ma et al. (2017) reported approximately 80% nitrogen removal in a two-stage denitratation/ANAMMOX-nitrification (anoxic/oxic) system fed with media containing both NH_4_^+^ and sodium acetate. In their setup, nitrification process occurred in the second stage in which NO_3_^−^ was generated-and-recycled back to the first stage where denitratation and ANAMMOX processes occurred. Based on the performance, it was reported that a C/N ratio of about 2.6 led to 80% NRE. However, the observed COD requirements were higher than the theoretical 1.14, highlighting the challenges with minimization of COD losses during aeration. In addition, metagenomic analysis revealed higher hits for nitrate reductase compared to nitrite reductase, an indication of faster rate of NO_2_^−^ production from NO_3_^−^ reduction compared to its depletion. Furthermore, it was reported that the dominant microorganisms in the anoxic biofilms were *Ca. Jettenia* spp. (17.83%), *Ca. Kuenenia* spp. (2.62%), and *Thauera* (5.27%).

In another study, Kamei et al. (2022) reported approximately 64% accumulation of NO_2_^−^ in a system supplied with H_2_ and NO_3_^−^ (Table 5). However, some of the supplied NO_3_^−^ was not converted to NO_2_^−^ and could not be accounted for in the effluent, an indication that some of the NO_2_^−^ was oxidized further leading to its removal. In addition, dominance of bacteria within the unidentified genus of *Comamonadaceae* was reported (Kamei et al., 2022). Furthermore, an optimal H_2_ supply rate of 0.7 (7 min on/3 min off at 15 ml/min flow rate) was reported, while higher and lower rates lead to a decrease in NO_2_^−^ accumulation.

Studies have reported different C/N ratios in systems incorporating PD and ANAMMOX (Table 5). In all the reported studies, the C/N ratios are higher than the theoretical 1.14 g-COD/g-N, an indication of losses through aerobic oxidation. However, the observed values are still lower than that for full denitrification whose C/N ratios are >5. Furthermore, the efficiencies of PD vary in different systems, possibly due to the variation of the microorganisms based on the available carbon sources as previously suggested by Du et al. (2017), or due to some other unknown factors.

Nitrogen removal in DEAMOX systems is comparable to that of PN/A systems (Tables 4, 5). In both systems, process performance has been shown to be influenced by many factors, among which are the reactor configuration and the efficiencies of PD and nitritation (Lackner et al., 2014a; Le et al., 2019b). NREs ranging from 41% to 99.9% were reported in the reviewed articles in DEAMOX systems and NRR was only reported from a single study (Table 5). The factors influencing performance of this process are discussed in “Discussion” together with the factors affecting the other processes.

## Biological degradation of cyanide

Some wastewater streams contain cyanide in high concentrations (Table 2). Its removal through biological degradation is favored to physico-chemical processes since this process does not generate harmful chemicals (Akcil et al., 2003; Barakat et al., 2004). Indeed, some bacterial species have been reported to be able to degrade complexed and free cyanide as well as thiocyanide (SCN^−^) to ammonia (NH_3_) and carbonate (Equations 5 and 6). *Pseudomonas spp.* and *Burkholderia cepacia* are some of the identified microorganisms with this capability (Akcil et al., 2003; Gurbuz et al., 2009).


(5) $$2{\mathrm{CN}}^{-}+4H_{2}O+O_{2}\to 2{\mathrm{HCO}}_{3}^{-}+2{\mathrm{NH}}_{3}$$

(6) $${\mathrm{SCN}}^{-}+2H_{2}O+\frac{5}{2}O_{2}\to {\mathrm{SO}}_{4}^{2-}+{\mathrm{NH}}_{3}+{\mathrm{HCO}}_{3}^{-}$$

Some fungi including *Fusarium solani* can also degrade cyanide in a two-step process under alkaline conditions (Dumestre et al., 1997; Equations 7 and 8). However, the degradation rate of cyanide is reportedly slow (*ca.* 1 mmol/h-mg dry cells), possibly due to slow hydrolysis of cyanide at pH > 9. *Cyanide hydratase* and *Rhodanese enzymes* isolated from *Trichoderma* spp. can catabolize cyanide leading to formation of HCONH_2_ and SCN^−^, respectively (Ezzi and Lynch, 2002). On the other hand, *Pseudomonas fluorescens* and *Pseudomonas putida* can aerobically oxidize cyanide to CO_2_ and NH_3_ using *cyanide oxidase* enzyme (Suh et al., 1994; Chapatwala et al., 1998).


(7) $$\mathrm{HCN}+H_{2}O\to {\mathrm{HCONH}}_{2}$$

(8) $${\mathrm{HCONH}}_{2}+H_{2}O\to \mathrm{HCOOH}+{\mathrm{NH}}_{3}$$

Plants also possess enzymes for cyanide detoxification (*betacyanoalanine synthase*) which can convert cyanide to non-toxic asparagine (C_4_H_8_N_2_O_3_; Trapp et al., 2003). However, there exists threshold concentrations beyond which cyanide is toxic to plants. For instance, willows can only survive for a few days at 20 mg-CN/L in a hydroponic system, and at 50 mg-CN/L in a sand irrigated system. On the contrary, algal species *Scenedesmus obliquus* have been reported to degrade cyanide concentrations as high as 400 mg/L even without prior adaptation, while *Chlorella* sp. were reported to be able to degrade *ca.* 86% of 100 mg/L solution in a 25-h period (Gurbuz et al., 2004). Aggregation of *Scenedesmus obliquus* cells was observed at 400 mg/L cyanide concentration, possibly as a means of minimizing the impact of cyanide on the cells. However, the mechanisms of algal degradation of cyanide still needs further investigations for better understanding.

Enzymatic degradation of cyanide to NH_4_^+^ and formate (HCOO^−^) in a single step has also been demonstrated (Equation 9; Basheer et al., 1992). These enzymes are developed from particular fungi and bacteria such as *Fusarium lateritium, Bacillus pumilus C1* and *Alcaligenes denitrificans*, respectively (Ingvorsen et al., 1991; Basheer et al., 1992; Meyers et al., 1993). Ingvorsen et al. (1991) reported near complete removal of cyanide by *Alcaligenes xylosoxidans* subsp. *denitrificans* in a medium containing approximately 25 ppm (parts per million) cyanide. Basheer et al. (1992) also reported near complete removal of cyanide by enzyme developed from *Alcaligenes denitrificans.* Other microorganisms including *Thiobacillus thioparus. Pseudomonas stutzeri,* etc. possess degrading abilities for CN^−^ or its derivatives (Katayama et al., 1992; Meyers et al., 1993; Watanabe et al., 1998).


(9) $$\mathrm{HCN}+2H_{2}O\to {\mathrm{HCOO}}^{-}+{\mathrm{NH}}_{4}^{+}$$

Chen et al. (2008) reported that immobilized cells of *Klebsiella oxytoca* could tolerate wider range of pH compared to suspended cells. In addition, immobilized cells were found to be less affected at higher CN^−^ concentrations (≥3 mM) compared to suspended cells. Indeed, the removal rates by suspended cells decreased from 49 to 12% when KCN concentration increased from 3 to 6 mM, while that of the cells immobilized in alginate and cellulose triacetate decreased from 59 to 20% and 60 to 26%, respectively (Chen et al., 2008). It is possible that the morphological changes and adsorption in the biofilms softened the impact of high CN^−^ concentrations on immobilized cells. Similar findings were reported by Babu et al. (1992) in an experimental set up containing 400 mg/L NaCN and a culture of *Pseudomonas putida* immobilized in calcium alginate beads. Overall, biological degradation of CN^−^ at large scale is feasible (Table 6). However, its degradation systems need to be combined with other NH_4_^+^-oxidizing processes process such as PN/A, DEAMOX or nitritation-denitritation for complete nitrogen removal.

**Table 6:** Biodegradation of cyanide by fungi, bacteria and enzyme-based catalysts.

	Microorganism/Enzyme	CN^−^ conc. (mg/L)	Temperature (°C)	pH	Cyanide removal	References
1	*Bacillus pumilus* C1		37	7.8–8^a^		Meyers et al. (1993)
2	*Trichoderma* spp.	40	25	8	-	Ezzi and Lynch (2002)
3	*Arthrospira maxima*	50–100	N/A^b^	N/A^b^	inhibited	Gurbuz et al. (2004)
4	*Chlorella* sp.		N/A^b^	N/A^b^	86%	
5	*Scenedesmus obliquus*		30	10	99%	
6	*Pseudomonas putida*	100	25^a^	7.5^a^	20%–94%	Chapatwala et al. (1998)
7	*Klebsiella oxytoca*	26–156	30	7	12–91	Chen et al. (2008)

a Optimal.

b Not applicable (not investigated).

## Bioelectrochemical processes

Coupling of biotic and abiotic processes for nitrogen removal has been previously investigated and documented (Nguyen et al., 2015; Deng et al., 2016; Sander et al., 2017). In most bioelectrochemical systems (BESs), anode and cathode chambers are separated by a proton exchange membrane, and the electrodes therein are connected by an electrical conductor (Nguyen et al., 2015). During operation, the electrons flowing from the anode are used to reduce oxidized nitrogen compounds such as NO_3_^−^ and NO_2_^−^ in the cathode (Sander et al., 2017). The electrons could be generated through biological activity in the anode such as oxidation of organic carbon material, or through the supply of electrical energy (Nguyen et al., 2015; Sander et al., 2017).

Nguyen et al. (2015) found that increasing the supplied voltage from 0.7 to 0.9 V led to an increase in NRE from approximately 18% to 43%, but beyond 0.9 V, there was no improvement in nitrogen removal. Similar findings were made by Clauwaert et al. (2007) in a MFC (microbial fuel cell) with biotic anode and cathode. A comparison of two BESs by Nguyen et al. (2015) in which one had a biotic anode and another one with abiotic anode found that the one with abiotic anode led to lower nitrogen removal (43%) compared to the one with biotic anode (75.4 ± 2.9%). The high NRE with biotic anode as reported by Nguyen et al. (2015) was achieved despite C/N ratio = 3 mg-COD/mg-NO_3_^−^-N), which is lower than for the conventional heterotrophic denitrification, highlighting the potential of this technology for treatment of low carbon (C/N ≤ 3) wastewater. Community analysis revealed that the bacterial consortia in biocathodes of BES with biotic anode differed from those with abiotic anode: species affiliated to *Shinella* sp., *Nitratireductor* sp. and *Dyella* sp. were dominated in BES with abiotic anode, while *Aeromonas* sp., *Pseudomonas* sp. and *Curtobacterium* sp. dominated in BES with biotic anode Nguyen et al. (2015). The observed variation in bacterial communities on biocathodes with the type of anode used still require further investigation to unravel the potential reasons behind these findings.

Transient accumulation of NO_2_^−^ is normally observed in BES, possibly due to slower NO_2_^−^-reduction compared to NO_3_^−^ in line with several previous reports (see “Denitrifying ammonium oxidation “; Park et al., 2006; Nguyen et al., 2015). In addition, accumulation of NH_4_^+^ has also been reported in these systems, an indication that dissimilatory nitrate reduction (DNR) could be occurring in BESs (Nguyen et al., 2015).

Most of the NRRs for BESs are low (<0.1 kg-N/m^3^-day), except for those reported by Sander et al. (2017), Feleke et al. (1998) and Isabel San-Martín et al. (2018) (Table 7). The systems reported by Sander et al. (2017) and Feleke et al. (1998) were up-flow columns, an indication that the motion of wastewater within the cells might have played a role in enhancing process kinetics, while nitrogen removal in the system reported by Isabel San-Martín et al. (2018) was suggested to have been undertaken by AMX because of the nature of its design. According to Sander et al. (2017), wastewater recirculation could have enhanced the transport of ions and gases from the surface of electrodes leading to the exposure of the surfaces once again.

**Table 7:** Performance of bioelectrochemical processes.

	Process description	Influent N concentrations (mg-N/L)	NRE (%)	NRR (kg-N/m^3^-day)	Remarks	References
1	BES (Abiotic anode—biocathode)	4.4–6.8	54–100	0.018–0.121	2 l up-flow column with recirculation units	Sander et al. (2017)
1	Abiotic anode—biocathode	50	18–43	0.00063–0.00156	Applied voltage: 0.7–1.1 V; 25°C	Nguyen et al. (2015)
2	Biotic anode—biocathode	50	75.4 ± 2.9	0.0024 ± 0.00035	Cell voltage variation between 0.1 V and 1 V; 25°C; C/N ratio = 3; 150 mg-COD/L CH_3_COONa	
3	Microbial fuel cell (MFC): biotic anode and cathode	2,615	-	0.08	1 g/L CH_3_COONa in the anodic section and KNO_3_ in cathodic section	Clauwaert et al. (2007)
4	Microbial electrolysis cell (biotic anode and cathode)	30.5	90–95^a^	0.01	Applied voltages were 0.4 V, 0.6 V and 0.8 V	Liang et al. (2021)
5	Electrochemical denitrification cell (abiotic anode and cathode)	30.5	57–62 ^a^	-	Abiotic conditions were applied	
6	H_2_	20	0–100	0.12^b^	Current supplied: −2 to 10 mA	Feleke et al. (1998)
7	biotic anode and cathode with S^2−^ addition in cathode chamber	100	100	-	400 ml system	Jianping et al. (2022)
8	Microbial electrolysis cell	30–1,400^c^	70	0.98^c^	150 l MFC; 1 V between anode and anode	Isabel San-Martín et al. (2018)

a For both Ti mesh and stainless steel mesh cathodes.

b Estimated from Figure 2 in the source file.

c Estimated from Figure 4 in the source file.

Conductivity of wastewater could also influence process performance (Sander et al., 2017; Liang et al., 2021). Since increased flow of wastewater could move the ions within the BESs, this could improve conductivity and lower its ohmic resistance (Sander et al., 2017). As reported by Sander et al. (2017), wastewater recirculation decreased the total resistance by approximately 13.6%, an indication that the transport of ions therein had an influence.

Energy consumption in BES was approximated by Sander et al. (2017) to be 0.027 ± 0.001 kWh/m^3^, which translated to 12.7–72.5 kWh/kg-N, which is over 5 times higher than for conventional activated sludge process. A huge part of the energy consumption was reported in the gap between the electrodes (*ca.* 13.5%). Therefore, the gap between the electrodes could thus be reduced to a minimum in order to minimize energy losses. However, precautions should be taken when reducing the gap so that oxygen does not diffuse into the cathode region as this would affect the bacterial activities, and promote the growth of aerobic microorganisms (Sander et al., 2017).

Addition of electron donors to the cathode chambers of BESs were demonstrated by Jianping et al. (2022) to enhance nitrogen removal. In addition, it was also reported that the system could generate an average output voltage of 450 mV lasting about 35 h. This thus indicates that BESs could also be utilized in power generation as a way of recovering the resources, and drive the wastewater treatment toward energy autarky.

## Autotrophic denitrification

Electron donors such as S, H_2_, sulfide (S^2−^), SCN^−^, thiosulfate (S_2_O_3_^2−^), sulfite (SO_3_^2−^), metallic elements and ions can be used by autotrophic denitrifiers (Cardoso et al., 2006; Di Capua et al., 2019; Statiris et al., 2021). The biomass yield of autotrophic denitrifiers is generally lower than for heterotrophic denitrifiers (Cui et al., 2019), which is advantageous because of less work (cost) in sludge handling. However, metallic elements and ions as well as S and its compounds generate harmful chemicals (Di Capua et al., 2019).

S^2−^ can exist in water as molecular hydrogen sulfide (H_2_S) or ionic hydrosulfide (HS^−^)/sulfide (S^2−^) depending on the prevailing conditions (Wiemann et al., 1998; Di Capua et al., 2019). S^2−^ presents serious environmental challenges because of its toxicity, corrosiveness and odor (Di Capua et al., 2019). In addition, S^2−^ is inhibitory to many microorganisms at elevated concentrations including to its consumers (autotrophic denitrifiers; Cardoso et al., 2006).

Stoichiometric reactions for autotrophic denitrification with complete and partial S^2−^ oxidation from NO_3_^−^ and NO_2_^−^ are presented in Equations 10 and 11 (Moraes et al., 2012). The extent of S^2−^ reduction is reportedly influenced by its concentration, with excess concentration leading to production of S according to Equation 17 (Cardoso et al., 2006). Although in some reports, S^2−^ oxidation to sulfate (SO_4_^2−^) has been suggested to be a two-step process in which S^2−^ is first oxidized to S, followed by S oxidation to SO_4_^2−^ (Cardoso et al., 2006), in others, it has been suggested that some bacteria oxidize S^2−^ to SO_4_^2−^ in a single step without S generation (Cui et al., 2019). According to Moraes et al. (2012), the two-step process is the preferred pathway for autotrophic S-utilizing denitrifiers, possibly because of lower electron requirements compared to its direct oxidation from S^2−^ to SO_4_^2−^. The formation of S as an intermediate product could lead to formation of white/yellowish color which subsides when S is subsequently consumed. In addition, intermediate production and subsequent consumption of S lead to fluctuation in alkalinity since S generation from S^2−^ lead to alkalinity production (Equation 17), while S oxidation to SO_4_^2−^ lead to alkalinity removal (Equation 12) (Doğan et al., 2012; Moraes et al., 2012; Di Capua et al., 2019). In comparison, NO_2_^−^ is reportedly consumed at a higher rate compared to NO_3_^−^, possibly because of its higher reactivity (Moraes et al., 2012).


(10) $$\begin{array}{c} {\mathrm{HS}}^{-}+1.23{\mathrm{NO}}_{3}^{-}+0.438{\mathrm{HCO}}_{3}^{-}+0.027{\mathrm{CO}}_{2}\\ +0.093{\mathrm{NH}}_{4}^{+}+0.573H^{+}\to 0.093C_{5}H_{7}O_{2}N\\ +{\mathrm{SO}}_{4}^{2-}+0.614N_{2}+0.866H_{2}O \end{array}$$

(11) $$\begin{array}{c} S^{2-}+2.26{\mathrm{NO}}_{2}^{-}+0.06{\mathrm{HCO}}_{3}^{-}+0.24{\mathrm{CO}}_{2}+0.06{\mathrm{NH}}_{4}^{+}\\ +2.26H^{+}\to 0.06C_{5}H_{7}O_{2}N+{\mathrm{SO}}_{4}^{2-}+1.13N_{2}+1.07H_{2}O \end{array}$$

(12) $$\begin{array}{l} 1.10S+{\mathrm{NO}}_{3}^{-}+0.76H_{2}O+0.4{\mathrm{CO}}_{2}+0.08{\mathrm{NH}}_{4}^{+}\\ \to 0.08C_{5}H_{7}O_{2}N+0.5N_{2}+1.1{\mathrm{SO}}_{4}^{2-}+1.28H^{+} \end{array}$$

Among the S compounds, S_2_O_3_^2−^ is consumed at a higher rate compared to S and S^2−^, possibly because it is readily available and non-toxic compared to S^2−^ which is toxic, and S which cannot be readily accessed by microorganisms because of mass transfer-related challenges (Cardoso et al., 2006). Notwithstanding the findings, S and S^2−^ have been successfully utilized as electron donors in denitrifying systems (Table 8). Indeed, pilot- and full-scale S-based autotrophic removal of NO_3_^−^ in wastewater was reported by Sahinkaya et al. (2014) in column bioreactors, as well as by Woo et al. (2022) in a A_2_O pilot-scale system. It is noteworthy that NO_3_^−^ reduction to N_2_ gas is a multi-step process that involves its reduction to NO_2_^−^, followed by further NO_2_^−^ reduction to N_2_ (Chung et al., 2014). In those steps, counter changes in the pH would occur, as NO_3_^−^ reduction would consume alkalinity, while NO_2_^−^ reduction consumes acidity (Chung et al., 2014 [Equations 13 and 14]). However, despite the counter changes, Chung et al. (2014) found that starting at a pH of approximately 8 in a batch experiment, the final pH was 7.8, an indication that not all the alkalinity was recovered in the final step. In addition, process operation at pH of 8 was reported to be optimal by Chung et al. (2014), in agreement with Pan et al. (2018).


(13) $$\begin{array}{c} S_{2}O_{3}^{2-}+3.1{\mathrm{NO}}_{3}^{-}+0.73H_{2}O+0.45{\mathrm{HCO}}_{3}^{-}+0.09{\mathrm{NH}}_{4}^{+}\\ \to 0.09C_{5}H_{7}O_{2}N+3.1{\mathrm{NO}}_{2}^{-}+2{\mathrm{SO}}_{4}^{2-}+1.64H^{+} \end{array}$$

(14) $$\begin{array}{c} S_{2}O_{3}^{2-}+2.07{\mathrm{NO}}_{2}^{-}+0.43H^{+}+0.45{\mathrm{HCO}}_{3}^{-}+0.09{\mathrm{NH}}_{4}^{+}\\ \to 0.09C_{5}H_{7}O_{2}N+2{\mathrm{SO}}_{4}^{2-}+1.03N_{2}+0.3H_{2}O \end{array}$$

**Table 8:** Performance of autotrophic denitrification systems.

	Electron donor	Influent N concentrations (mg-N/L)	NRE (%)	NRR (kg-N/m^3^-day)	Remarks	References
1	S^2−^	20	25–98	-	NO_3_^−^—fed; N/S = 0.9&1.7	Moraes et al. (2012)
2		20	95–100	0.009	NO_2_^−^—fed; N/S = 1.5&2.8	
3	S_2_O_3_^2−^	56	-	0.47	415 mgSO_4_^2−^produced	Cardoso et al. (2006)
4	S^2−^		-	0.10	87 mgSO_4_^2−^produced	
5	S		-	0.050	16 mgSO_4_^2−^produced	
6	S	30–60	30–100^b^	0.30	Sulphur/limestone (v/v) = 1–3	Sahinkaya et al. (2014)
7		25 ± 3	100	0.15	Sulphur/limestone (v/v) = 1	
8	H_2_	20–40	92–96	2.7–5.3^c^	Microporous membrane bioreactor	Mansell and Schroeder (2002)
9	S	29–31	79–98	0.05–0.36	10 L sulfoxidizing MBBR	Cui et al. (2019)
10	FeS_2_	27	57	0.080^d^	25–50 μm and 50–100 μm pyrite particles	Torrentó et al. (2010)
11	S_2_O_3_^2−^	60–80	-	0.058^e^	S_2_O_3_^2−^-driven denitritation	Qian et al. (2016)
12	H_2_	25–400	97	1.9–5.8	16.8 l pressurized reactor	Keisar et al. (2021)

a Approximated from Figure 4a in the source file.

b Estimated from Figure 4 in the source file.

c g-NO_3_^−^-N/m^2^-day.

d g-NO_3_^−^-N/kg-pyrite-day.

e Estimated from Figure 3c in the source file.

SCN^−^, which is present in considerable concentrations in gold mining and coking wastewaters, can be utilized as an electron donor in denitrifying systems (Equation 15; Pan et al., 2018). However, SCN^−^ leads to NH_4_^+^ production during its oxidation (Equation 15), which would require further nitritation (“Nitritation-denitritation”). Pan et al. (2018) suggested that SCN^−^ oxidation takes place in three steps leading to the formation of S as an intermediate product, which is subsequently removed in the final step (Equations 16–18).


(15) $$\begin{array}{c} {\mathrm{SCN}}^{-}+2{\mathrm{NO}}_{2}^{-}+1.9H^{+}+1.2H_{2}O\\ \to 0.10C_{5}H_{7}O_{2}N+{\mathrm{SO}}_{4}^{2-}+N_{2}+0.5{\mathrm{CO}}_{2}+0.90{\mathrm{NH}}_{4}^{+} \end{array}$$

(16) $$5{\mathrm{SCN}}^{-}+H_{2}O\to S^{2-}+{\mathrm{CO}}_{2}+{\mathrm{CO}}_{2}$$

(17) $${\mathrm{NO}}_{2}^{-}+1.5S^{2-}+4H^{+}\to 1.5S+0.5N_{2}+2H_{2}O$$

(18) $$2{\mathrm{NO}}_{2}^{-}+S\to {\mathrm{SO}}_{4}^{2-}+N_{2}$$

Some of the known sulfoxidizing denitrifiers include *Thiobacillus denitrificans, Paracoccus pantotropha, Paracoccus denitrificans, Paracoccus versutus*, *Thioploca* spp. and *Beggiatoa* spp. (Cardoso et al., 2006). Other reported sulfoxidisers include *Sulfurimonas denitrificans, Thiobacillus denitrificans* and *Thiohalobacter denitrificans* (Liang et al., 2021). Although some of these denitrifiers are strict chemolithoautotrophic, others are facultative. These denitrifiers are distributed across α-, β-, ε- and γ-*Proteobacteria* (Di Capua et al., 2019).

Feasibility of hydrogen-based autotrophic denitrification has also been demonstrated (Feleke et al., 1998; Mansell and Schroeder, 2002; Lee et al., 2010; Liang et al., 2021). In this process, denitrifiers use H_2_ as electron donor according to Equation 19 to reduce NO_3_^−^ to N_2_. H_2_, in turn, could be generated from purely chemical processes such as electrochemistry (“Biological degradation of cyanide”), or from biological sources (Liang et al., 2021).


(19) $$\begin{array}{c} 0.33NO_{3}^{-}+H_{2}+0.08CO_{2}+0.34H^{+}\\ \to 0.015C_{5}H_{7}NO_{2}+1.11H_{2}O+0.16N_{2} \end{array}$$

In a system supplied with H_2_ and fed with 22.5 mg-NO_3_^−^-N/L, Szekeres et al. (2002) found that *Ochrobactrum anthropi, Pseudomonas stutzeri, Paracoccus panthotrophus* and *Paracoccus denitrificans* were dominant, an indication that these bacteria were responsible for hydrogenotrophic denitrification. Other previously reported hydrogenotrophic denitrifiers include *Azospirillum brasilence, Rhizobium japonicum, Hydrogenophaga flava, Hydrogenophaga pseudopflava, Hydrogenophaga taeniospiralis and Ralstonia eutropha* (Mansell and Schroeder, 2002). Liang et al. (2021) also reported the dominance of Rhodocyclaceae, *Paracoccus* spp. and *Dethilobacter* spp. in a bioelectrochemical system in which the cathode chamber received H_2_ from the anode chamber.

Torrentó et al. (2010) demonstrated the reduction of NO_3_^−^ using pyrite (FeS_2_) as an electron donor in batch and flow-through experiments conducted using *Thiobacillus denitrificans*-dominated cultures. Although complete NO_3_^−^ removal was observed, low NRRs were reported (<0.080 g-NO_3_^−^-N/kg-pyrite-day). In addition, it was reported that process kinetics were influenced by grain sizes of pyrite with complete nitrogen removal in experiments with 25–50 μm particles being observed after 14 days, while longer (>14 days) periods were needed in experiments with 50–100 μm particles, probably due to mass transfer challenges. Despite the low NRRs, pyrite-driven process produces less SO_4_^2−^ compared to S-driven denitratation. Both iron (II) ions (Fe^2+^) and S anions donate electrons for NO_3_^−^ reduction to N_2_ and/or NO_2_^−^ according to Equations 20–22 (Torrentó et al., 2010):


(20) $$5{\mathrm{FeS}}_{2}+14{\mathrm{NO}}_{3}^{-}+4H^{+}\to 5{\mathrm{Fe}}^{2+}+2H_{2}O+7N_{2}+10{\mathrm{SO}}_{4}^{2-}$$

(21) $$10{\mathrm{Fe}}^{2+}+2{\mathrm{NO}}_{3}^{-}+12H^{+}\to 10{\mathrm{Fe}}^{3+}+6H_{2}O+N_{2}$$

(22) $$2{\mathrm{FeS}}_{2}+15{\mathrm{NO}}_{3}^{-}+7H_{2}O\to 2\mathrm{Fe}{\left(\mathrm{OH}\right)}_{3}+15{\mathrm{NO}}_{2}^{-}+4{\mathrm{SO}}_{4}^{2-}+8H^{+}$$

Nitrogen removal *via* NO_2_^−^ as opposed to NO_3_^−^ could be implemented as a way of minimizing electron donor demand as denitritation requires only 2.5 S/N (sulfur to nitrogen ratio), while denitratation requires approximately 5.1 S/N (Chung et al., 2014). Therefore, nitrogen removal *via* NO_2_^−^ would provide approximately 50% savings on electron donors. Approximately 40% savings on hydrogen would also be expected on removing nitrogen *via* NO_2_^−^ as opposed to NO_3_^−^ (Rezania et al., 2005).

The reported NRRs in autotrophic denitrification systems driven by S and its compounds range between 0.009–0.47 kg-N/m^3^-day (Table 8). These rates are small and it would be challenging to apply in high-strength systems that require higher denitrification rates in order to avoid handling challenges, particularly in relation to the equipment. Therefore, further improvements could be made in order to emerge as a robust process that can treat high-strength wastewaters. On the contrary, hydrogenotrophic denitrification conducted in pressurized vessels have been reported to have high NRRs (>1 kg-N/m^3^-day; Table 8). These rates are comparable to those of PN/A and nitritation-denitritation (Tables 3–5), an indication that these technology can be utilized at full-scale level provided that challenges with H_2_ handling can be overcome.

## Discussion

Several low carbon nitrogen removal processes have been developed and applied at pilot- and full-scale level (Supplementary Table S1 supplementary information). However, each of these processes is restricted by a number of factors influencing the NREs and NRRs. In addition, efficient generation and utilization of electron donors and acceptors by different microorganisms is always a challenge, as regulatory measures need to be put in place to limit the activities of competing microorganisms that would otherwise present competition. Furthermore, operating conditions such as pH/alkalinity, temperature, DO, ionic strength, etc. impact on the performance of the BNR systems and would require regulation.

Nitritation-denitritation requires theoretical C/N ratios of 1.71 g-COD/g-N, which is higher than that of DEAMOX that requires 1.14 g-COD/g-N. Since DEAMOX requires only about half of influent NH_4_^+^ to be oxidized to NO_3_^−^ with subsequent reduction to NO_2_^−^ through PD, the actual demand for oxygen in this process is much less than that of partial nitritation-denitritation. Based on these facts, DEAMOX would thus be cheaper than nitritation-denitritation. However, comparisons of the actual COD requirements in the two processes in different studies have revealed that there has been comparable COD consumption in both processes (Tables 3 and 5). This highlights the inefficiencies in COD consumption, with poorer utilization in DEAMOX systems compared to nitritation-denitritation systems. It is possible that the COD is lost through oxidation by aerobic heterotrophic bacteria when the systems are aerated. Therefore, proper process control units need to be put in place to enhance COD utilization in these systems. This could include sequencing of operations to allow dosing of COD during anaerobic periods in order to limit losses through aerobic oxidation. It could also be possible to have a multi-stage system that could allow COD dosing in anaerobic/anoxic tanks only, and aeration in the respective tanks as was demonstrated by Le et al. (2019b). Despite the limitations, nitritation-denitritation has been applied at pilot- and full-scale systems (Supplementary Table S1) since the process kinetics are favorable (>0.5 kg-N/m^3^-day; Table 3).

Bioelectrochemical processes (in BESs) are complex in nature and would require personnel with technical skills to operate. The reported NRRs in most of the studies presented in Table 7 are low (≤0.12 kg-N/m^3^-day), except those reported by Isabel San-Martín et al. (2018) in a 150 l pilot-scale system in which AMX was suggested to have co-existed with other bacteria. Therefore, further discussion of the findings from this study within the framework of BESs will not be made as it would lead to misrepresentation.

Performance of BESs are limited by low water conductivities, which increase internal resistance, in turn, leading to limitation in the rates of denitrification, and an increase in energy losses (Sander et al., 2017; Liang et al., 2021). The operation of bioelectrochemical systems thus require electron donor and acceptor sites to be in close proximities in order to reduce energy wastage (Sander et al., 2017), which limit their application in full-scale systems (Liang et al., 2021). In addition, the overall construction of the bioelectrochemical cells is complex as it requires the integration of electrochemistry with biological activities. Furthermore, NH_4_^+^ generation (possibly through dissimilatory NO_3_^−^ reduction) reduce the NRE of bioelectrochemical processes. Further research could thus provide solutions to the challenges still limiting BES to pilot-scale and laboratory-scale applications in its current state of development (Supplementary Table S1).

BESs could be advantageous for hydrogenotrophic denitrification since H_2_ is generated *in situ* which enhances its delivery to denitrifiers (Sander et al., 2017). Alternatively, microorganisms might utilize electrons direction without the need for H_2_ generation, which could improve process efficiency and kinetics. Generation and utilization of H_2_ at the cathode could also improve efficiency of utilization by the microorganisms, unlike in systems where H_2_ is supplied from external sources as its poor solubility in wastewater could lead to low utilization efficiencies (Sander et al., 2017).

Autotrophic denitrification appears to have higher NRRs compared to bioelectrochemical processes (Tables 7 and 8). However, the NRRs for S-based processes are still low (<0.47 kg-N/m^3^-day), and its application would require huge reactors in large municipal systems. This notwithstanding, full-scale autotrophic denitrification driven by S and its compounds have been developed (Sahinkaya et al., 2014; Supplementary Table S1). Moreover, S compounds are present in different forms in anaerobic wastewater treatment systems as well as in many other streams including tannery wastewater (Wiemann et al., 1998), and its utilization for nitrogen removal is thus necessary as a cost-saving measure instead of sourcing external carbon supply. For instance, Wiemann et al. (1998) reported S^2−^, organic S and NH_4_^+^ concentrations of 360 mg/L, 270 mg/L and 385 mg-N/L in tannery wastewater, respectively, meaning that complete removal of nitrogen would require approximately 1101.1 mg-COD/L (based on 2.86*385) that could be obtained from the S^2−^ and organic S (~1,125 mg-COD/L), assuming efficient utilization. Therefore, autotrophic denitrification driven by elemental S and its compounds present viable alternative to the currently applied heterotrophic denitrification and anammox-mediated processes, and could be further optimized for a widespread application. However, generation of SO_4_^2−^ in these processes could limit their widespread application as they have inhibitory effects on several biological processes including desulfoxidization (Chung et al., 2014). S^2−^ has been associated with bad odor and corrosiveness (Di Capua et al., 2019), and this could influence its selection as an electron donor in denitrification systems. However, since S^2−^ is present in biogas scrubbing columns (Vu et al., 2022), denitrifying systems could be engineered to utilize the electron donors therein as a cost-cutting measure.

Hydrogenotrophic denitrification was demonstrated by Keisar et al. (2021) and Mansell and Schroeder (2002) in membrane and pressurized bioreactors, respectively, to have high NRRs (1.9–5.8 kg-N/m^3^-day). The reported NRRs are comparable to those previously reported for anammox-mediated processes and nitritation-denitritation (Tables 3–5). However, pressurization of vessels and membrane lumens consume extra electrical energy besides that which is consumed in the operation of pumps and other devices, and could increase the cost of treatment process to unsustainably high levels. Handling H_2_ requires care, and its usage could thus be further limited by the challenges faced in its handling. Nonetheless, despite these challenges, hydrogenotrophic denitrification completely remove nitrogen unlike PN/A which generate about 11% NO_3_^−^ per NH_4_^+^ consumed. Compared to denitrification driven by metallic ions, elemental S and its compounds, hydrogenotrophic denitrification does not generate any environmentally harmful chemicals.

Different microorganisms and plants have been shown to possess varying capacities for CN^−^ removal (“Biological degradation of cyanide”). Some specific enzymes, in particular, have been demonstrated to degrade CN^−^ (and SCN^−^), leading to NH_3_ generation. However, the NH_3_ generated in the degradation would require removal in downstream systems in order to avert its negative impact on the environment. Therefore, coupling CN^−^ degradation with NH_4_^+^ removal processes such as nitritation-denitritation and ANAMMOX-mediated processes would be imperative for successful nitrogen removal. Alternatively, SCN^−^-containing wastewater could be treated with NO_3_^−^-containing wastewater so as to allow autotrophic denitrifiers to utilize SCN^−^ as electron donor in the reduction of nitrate (“Autotrophic denitrification”). This approach would eliminate the need for separate treatment of SCN^−^ and NO_3_^−^ in the respective streams. On the other hand, the growth of fungi in wastewater treatment systems is limited, and their impact on nitrogen removal might be insignificant. Similarly, the nitrogen removal kinetics of plants and microalgae might be lacking behind those of bacteria, and their use might need further analyses.

Inhibition of bacterial activities by CN^−^ at concentrations 0.2–1.0 mg/L has been reported in several studies, and its fate in wastewater treatment systems has been adequately addressed (Wild et al., 1994; Dash et al., 2009; Kim et al., 2011; Kapoor et al., 2016). The report by Landkamer et al. (2015) demonstrated co-removal of CN^−^, NO_3_^−^ and NH_4_^+^, in a system containing mixed bacterial culture collected from heap leach sediment and water. From the study, it was suggested that CN^−^ was hydrolysed according to Equation 8 by native bacteria that were present in the heap leach sediments and water. Formate produced in such a reaction would have likely been utilized as electron donor in partial reduction of NO_3_^−^ to NO_2_^−^. The NO_2_^−^, in turn, would have then been used as electron acceptor in the anammox process, leading to NH_4_^+^ oxidation to nitrogen gas. The growth of AMX in an environment containing *ca.* 15 mg/L CN^−^ as suggested by Landkamer et al. (2015) contradicts the findings of Huang and Hogsett (2011) who found that anammox specific activities dropped by 50% at CN^−^ concentrations of just 0.05 mg/L during the first 2 days of incubation, although 80% of the performance was recovered thereafter. This suggests that AMX present in the culture used by Landkamer et al. (2015) could have adapted to the presence of high (15 mg/L) CN^−^ concentrations prior to the study as the culture was obtained from an environment containing high CN^−^ concentrations. Adaptation of AOB to high (≥ 10 mg/L) CN^−^ concentrations has also been demonstrated previously (Do et al., 2008). however, the findings by Do et al. (2008) contradicts Kapoor et al. (2016) who concluded that concentrations higher than 0.2 mg/L could inhibit AOB activities. In another study, Kim et al. (2011) reported that denitrifying bacteria could tolerate CN^−^ concentrations >40 mg/L, and that NOB are more sensitive to CN^−^ than AOB, which indicate that the impact of CN^−^ on different microorganisms could be different, and the inhibitory concentrations could vary with cultures. Further studies using advanced molecular techniques would thus be desirable in order to get a better understanding of the response by different microbial consortia to CN^−^. Nitrogen labeling could also assist in understanding the nitrogen transformation pathways in biological systems. Similarly, inhibition by SCN^−^ could also be further explored as varying concentrations have been reported for different cultures (Kim et al., 2011; Oshiki et al., 2018; Yu et al., 2020).

Low-cost and high efficiencies associated with PN/A has led to increasing interest in the technology (Lackner et al., 2014a). The benefits stem from reduced requirements for oxygen, low-sludge production, and elimination of COD requirements. With this process, it is possible to remove >80% of influent nitrogen (Table 4). However, since about 11% of influent NH_4_^+^ is converted to NO_3_^−^ in PN/A process, coupling this process with NO_3_^−^-reducing processes such as denitrification could still be necessary for complete nitrogen removal as was demonstrated by Xie et al. (2017). In such a case, the COD requirements would be *ca.* 0.3 g-COD/g-NH_4_^+^-N. In municipal systems, however, the PN/A effluent can be pumped to mainstream systems that contain high levels of COD, eliminating the need for external carbon (Abma et al., 2010; Wett et al., 2015). Compared to denitrification driven by metallic ions and compounds of S, PN/A does not generate any harmful chemicals. Moreover, in many full-scale systems, PN/A process has been applied to treat mainly landfill leachate and sidestream wastewaters with satisfactory performance without the need for complicated process configurations similar to that of hydrogenotrophic denitrification that require pressurized vessels. Wastewater treatment without external carbon supply minimizes operational costs and simplifies process configuration. Lower sludge production in PN/A process compared to nitritation-denitritation because PN/A process is carried out by autotrophic bacteria could further reduce the operational costs of the process.

The challenges affecting widespread application of PN/A include the sensitivity of the AMX to operating and environmental conditions, as well as the slow growth rates (doubling time: 2.1–11 days) (Van Der Star et al., 2007; Fernández et al., 2008; Zhang et al., 2017). As a result, other bacteria such as NOB and NO_2_^−^-reducing heterotrophic bacteria could easily out-compete AMX if proper control strategies are not installed. Regulation of C/N ratios is normally applied in order to control the growth of NO_2_^−^-reducing heterotrophic bacteria, while maintenance of low DO concentration, intermittent aeration, SRT/HRT control, FA and FNA inhibition is applied to control NOB growth in PNA systems (see “Nitritation-denitritation”). However, control of *Nitrospira*-affiliated NOB is challenging because they can grow under limited substrate and DO conditions such as in the biofilms (Constantine et al., 2016; Park et al., 2017b). According to Wett et al. (2013), NOB have longer enzymatic lag-phase compared to NOB. Therefore, application of intermittent aeration in PN/A systems could limit their growth as was demonstrated by Regmi et al. (2014). FA and FNA inhibition of NOB could also be exploited in PN/A systems compared to their higher sensitivity compared to AOB and AMX (see “Nitritation-denitritation”) (Anthonisen et al., 1976; Dapena-Mora et al., 2007; Fernández et al., 2012; Jaroszynski et al., 2012). Based on the data presented by Lackner et al. (2014a) and Bowden et al. (2015), PN/A has received wide acceptance as an alternative to nitrification/denitrification.

The application of DEAMOX under mainstream conditions has been demonstrated previously (Table 5). However, its application under sidestream conditions might not be attractive, as COD would have to be supplied from external sources. Therefore, in this regard, PN/A would be preferable to DEAMOX in sidestream processes. Compared to nitritation-denitritation, the COD requirements in DEAMOX is less since only approximately half of NH_4_^+^ would need to be oxidized to NO_3_^−^ before its PD to NO_2_^−^. The oxidation of only approximately half of NH_4_^+^ would also translate to about half of aeration costs in DEAMOX compared to nitritation-denitritation. The theoretical COD requirements for DEAMOX is approximately 0.57 g-COD/g-NH_4_^+^-N (assuming half of NH_4_^+^ is nitrified), which is lower than for nitritation-denitritation (*ca.* 1.71 g-COD/g-NH_4_^+^-N). Despite the huge potential of this technology, its highest level of application is at pilot-scale level (Le et al. 2019a,b; Supplementary Table S1).

## Recommendations for future studies

The following recommendations are made for future studies:

1. Investigate the factors triggering DNR in BES
2. Study and develop processes for improving H_2_ utilization and solubility in hydrogenotrophic denitrification systems
3. Investigate the methods for enhancing conductivity in BESs in order to minimize energy losses and enhance process performance
4. Couple autotrophic denitrification with heterotrophic denitrification as a way of minimizing generation of harmful chemicals
5. Optimize nitritation-denitritation and DEAMOX to enhance COD consumption efficiency

## Conclusion

Several low-carbon (C/N ≤ 3) nitrogen removal processes have been developed, including nitritation-denitritation, anammox-mediated processes, bioelectrochemical processes and autotrophic denitrification. Bioelectrochemical processes are limited by low conductivities of wastewater and are characterized by low NRRs, which limit their application in large-scale systems. There is inefficient consumption of COD in DEAMOX and nitritation-denitritation processes. Autotrophic denitrification driven by S and its compounds has moderate NRRs (<0.47 kg-N/m^3^-day) and thus requires further improvements in order to emerge as a viable alternative to anammox-mediated processes and nitritation-denitritation. Incorporation of NO_3_^−^-removal processes such as DEAMOX and autotrophic denitrification in PN/A systems could lead to complete nitrogen removal. It is necessary to couple enzymatic degradation of CN^−^ and SCN^−^ with NH_4_^+^-oxidation processes such as PN/A and nitritation-denitritation for a successful nitrogen removal. H_2_-deriven autotrophic denitrification has high NRRs (>1 kg-N/m^3^-day) and could substitute anammox-mediated processes and nitritation-denitritation. However, its application requires incorporation of pressurized vessels in process lines. Autotrophic denitrification driven by metallic ions as well as S and its compounds generate harmful chemicals which necessitate downstream removal to avert negative impact on the environment. Overall, anammox-mediated nitrogen removal processes present the best alternatives to nitrification/denitrification in terms of COD demand, simplicity and process performance.

## Author contributions

KK: conceptualization, investigation, formal analysis, writing—original draft. PZ: investigation, writing—original draft. FB: resources, supervision, funding acquisition. SK: conceptualization, resources, writing—review and editing, supervision, funding acquisition. All authors contributed to the article and approved the submitted version.

## Funding

The project is funded by the Water Research Commission of South Africa (grant C2019/2020-00103).

## Conflict of interest

The authors declare that the research was conducted in the absence of any commercial or financial relationships that could be construed as a potential conflict of interest.

## Publisher’s note

All claims expressed in this article are solely those of the authors and do not necessarily represent those of their affiliated organizations, or those of the publisher, the editors and the reviewers. Any product that may be evaluated in this article, or claim that may be made by its manufacturer, is not guaranteed or endorsed by the publisher.

## References

[ref1] Abeysiriwardana-Arachchige I. S. A. Munasinghe-Arachchige S. P. Delanka-Pedige H. M. K. Nirmalakhandan N. (2020). Removal and recovery of nutrients from municipal sewage: algal vs. conventional approaches. Water Res. 175:115709. doi: 10.1016/j.watres.2020.115709, PMID: 32213371

[ref2] Abma W. R. Driessen W. Haarhuis R. Van Loosdrecht M. C. M. (2010). Upgrading of sewage treatment plant by sustainable and cost-effective separate treatment of industrial wastewater. Water Sci. Technol. 61, 1715–1722. doi: 10.2166/wst.2010.977, PMID: 20371929

[ref3] Adams M. Xie J. Kabore A. W. J. Chang Y. Xie J. Guo M.. (2022). Research advances in anammox granular sludge: a review. Crit. Rev. Environ. Sci. Technol. 52, 631–674. doi: 10.1080/10643389.2020.1831358

[ref4] Akcil A. Karahan A. G. Ciftci H. Sagdic O. (2003). Biological treatment of cyanide by natural isolated bacteria (pseudomonas sp.). Miner. Eng. 16, 643–649. doi: 10.1016/S0892-6875(03)00101-8

[ref5] Albina P. Durban N. Bertron A. Albrecht A. Robinet J.-C. Erable B. (2019). Influence of hydrogen electron donor, alkaline pH, and high nitrate concentrations on microbial denitrification: a review. Int. J. Mol. Sci. 20:5163. doi: 10.3390/ijms20205163, PMID: 31635215 PMC6834205

[ref6] Anthonisen A. C. Loehr R. C. Prakasam T. Srinath E. (1976). Inhibition of nitrification by ammonia and nitrous acid. J. Water Pollut. Control Fed. 48, 835–852. 948105

[ref7] Awolusi O. O. Enitan A. M. Kumari A. Bux F. (2015). Nitrification efficiency and community structure of municipal activated sewage sludge. Int. J. Environ. Chem. Ecol. Geol. Geophys. Eng. 9, 996–1003.

[ref8] Azari M. Walter U. Rekers V. Gu J.-D. Denecke M. (2017). More than a decade of experience of landfill leachate treatment with a full-scale anammox plant combining activated sludge and activated carbon biofilm. Chemosphere 174, 117–126. doi: 10.1016/j.chemosphere.2017.01.123, PMID: 28160675

[ref9] Babu G. R. V. Wolfram J. H. Chapatwala K. D. (1992). Conversion of sodium cyanide to carbon dioxide and ammonia by immobilized cells of pseudomonas putida. J. Ind. Microbiol. 9, 235–238. doi: 10.1007/BF01569629 9523454

[ref10] Barakat M. A. Chen Y. T. Huang C. P. (2004). Removal of toxic cyanide and cu(II) ions from water by illuminated TiO2 catalyst. Appl. Catal. B Environ. 53, 13–20. doi: 10.1016/j.apcatb.2004.05.003

[ref11] Basheer S. Kut Ö. M. Prenosil J. E. Bourne J. R. (1992). Kinetics of enzymatic degradation of cyanide. Biotechnol. Bioeng. 39, 629–634. doi: 10.1002/bit.260390607 18600992

[ref12] Bernat K. Kulikowska D. Godlewski M. (2016). Crude glycerol as a carbon source at a low COD/N ratio provides efficient and stable denitritation. Desalin. Water Treat. 57, 19632–19641. doi: 10.1080/19443994.2015.1109555

[ref13] Blaszczyk M. (1993). Effect of medium composition on the Denitrification of nitrate by Paracoccus denitrificans. Appl. Environ. Microbiol. 59, 3951–3953. doi: 10.1128/aem.59.11.3951-3953.1993, PMID: 16349097 PMC182557

[ref14] Bowden G. Stensel H. D. Tsuchihashi R. (2015). Technologies for Sidestream nitrogen removal. Water Intell. 15:9781780407890. doi: 10.2166/9781780407890

[ref15] Cao S. Zhou Y. (2019). New direction in biological nitrogen removal from industrial nitrate wastewater via anammox. Appl. Microbiol. Biotechnol. 103, 7459–7466.doi: 10.1007/s00253-019-10070-3 31388729

[ref16] Cardoso R. B. Sierra-Alvarez R. Rowlette P. Flores E. R. Gómez J. Field J. A. (2006). Sulfide oxidation under chemolithoautotrophic denitrifying conditions. Biotechnol. Bioeng. 95, 1148–1157. doi: 10.1002/bit.21084, PMID: 16807929

[ref17] Cecconet D. Sabba F. Devecseri M. Callegari A. Capodaglio A. G. (2020). In situ groundwater remediation with bioelectrochemical systems: a critical review and future perspectives. Environ. Int. 137:105550. doi: 10.1016/j.envint.2020.105550, PMID: 32086076

[ref18] Chapatwala K. D. Babu G. R. V. Vijaya O. K. Kumar K. P. Wolfram J. H. (1998). Biodegradation of cyanides, cyanates and thiocyanates to ammonia and carbon dioxide by immobilized cells of pseudomonas putida. J. Ind. Microbiol. Biotechnol. 20, 28–33. doi: 10.1038/sj.jim.2900469, PMID: 9523454

[ref19] Chen C. Y. Kao C. M. Chen S. C. (2008). Application of Klebsiella oxytoca immobilized cells on the treatment of cyanide wastewater. Chemosphere 71, 133–139. doi: 10.1016/j.chemosphere.2007.10.058, PMID: 18082868

[ref20] Chen Z. Zheng X. Chen Y. Wang X. Zhang L. Chen H. (2020). Nitrite accumulation stability evaluation for low-strength ammonium wastewater by adsorption and biological desorption of zeolite under different operational temperature. Sci. Total Environ. 704:135260. doi: 10.1016/j.scitotenv.2019.135260, PMID: 31780159

[ref21] Christensson M. Ekstrom S. Andersson Chan A. Le Vaillant E. Lemaire R. (2013). Experience from start-ups of the first ANITA Mox plants. Water Sci. Technol. 67, 2677–2684. doi: 10.2166/wst.2013.156, PMID: 23787303

[ref22] Chung J. Amin K. Kim S. Yoon S. Kwon K. Bae W. (2014). Autotrophic denitrification of nitrate and nitrite using thiosulfate as an electron donor. Water Res. 58, 169–178. doi: 10.1016/j.watres.2014.03.071, PMID: 24755301

[ref23] Claros J. Serralta J. Seco A. Ferrer J. Aguado D. (2012). Real-time control strategy for nitrogen removal via nitrite in a SHARON reactor using pH and ORP sensors. Process Biochem. 47, 1510–1515. doi: 10.1016/j.procbio.2012.05.020

[ref24] Clauwaert P. Rabaey K. Aelterman P. De Schamphelaire L. Pham T. H. Boeckx P.. (2007). Biological Denitrification in Microbial Fuel Cells. Environ. Sci. Technol. 41, 3354–3360. doi: 10.1021/es062580r 17539549

[ref25] Constantine T. Sandino J. Houweling D. Stephan S. Yin H. Nielsen P. (2016). Incorporating leading edge mainstream Deammonification into full-scale advanced BNR facilities. Proc. Water Environ. Fed. 2016, 1007–1018. doi: 10.2175/193864716819714735

[ref26] Cui Y.-X. Biswal B. K. Van Loosdrecht M. C. M. Chen G.-H. Wu D. (2019). Long term performance and dynamics of microbial biofilm communities performing sulfur-oxidizing autotrophic denitrification in a moving-bed biofilm reactor. Water Res. 166:115038. doi: 10.1016/j.watres.2019.115038, PMID: 31505308

[ref27] Daigger G. T. (2014). Oxygen and carbon requirements for biological nitrogen removal processes accomplishing nitrification, Nitritation, and Anammox. Water Environ. Res. 86, 204–209. doi: 10.2175/106143013X13807328849459, PMID: 24734468

[ref28] Dapena-Mora A. Fernández I. Campos J. L. Mosquera-Corral A. Méndez R. Jetten M. S. M. (2007). Evaluation of activity and inhibition effects on Anammox process by batch tests based on the nitrogen gas production. Enzym. Microb. Technol. 40, 859–865. doi: 10.1016/j.enzmictec.2006.06.018

[ref29] Dash R. R. Gaur A. Balomajumder C. (2009). Cyanide in industrial wastewaters and its removal: a review on biotreatment. J. Hazard. Mater. 163, 1–11. doi: 10.1016/j.jhazmat.2008.06.051, PMID: 18657360

[ref30] Daverey A. Su S.-H. Huang Y.-T. Chen S.-S. Sung S. Lin J.-G. (2013). Partial nitrification and anammox process: a method for high strength optoelectronic industrial wastewater treatment. Water Res. 47, 2929–2937. doi: 10.1016/j.watres.2013.01.028, PMID: 23548564

[ref31] Deng S. Li D. Yang X. Zhu S. Xing W. (2016). Advanced low carbon-to-nitrogen ratio wastewater treatment by electrochemical and biological coupling process. Environ. Sci. Pollut. Res. 23, 5361–5373. doi: 10.1007/s11356-015-5711-0, PMID: 26564190

[ref32] Di Capua F. Pirozzi F. Lens P. N. L. Esposito G. (2019). Electron donors for autotrophic denitrification. Chem. Eng. J. 362, 922–937.doi: 10.1016/j.cej.2019.01.069

[ref33] Dimitrova I. Dabrowska A. Ekström S. (2020). Start-up of a full-scale partial nitritation-anammox MBBR without inoculum at Klagshamn WWTP. Water Sci. Technol. 81, 2033–2042. doi: 10.2166/wst.2020.271, PMID: 32666956

[ref34] Do H. Lim J. Shin S. G. Wu Y.-J. Ahn J.-H. Hwang S. (2008). Simultaneous effect of temperature, cyanide and ammonia-oxidizing bacteria concentrations on ammonia oxidation. J. Ind. Microbiol. Biotechnol. 35, 1331–1338. doi: 10.1007/s10295-008-0415-9, PMID: 18712557

[ref35] Dobbeleers T. Caluwé M. Dockx L. Daens D. D'aes J. Dries J. (2020). Biological nutrient removal from slaughterhouse wastewater via nitritation/denitritation using granular sludge: an onsite pilot demonstration. J. Chem. Technol. Biotechnol. 95, 111–122. doi: 10.1002/jctb.6212

[ref36] Doğan E. C. Türker M. Dağaşan L. Arslan A. (2012). Simultaneous sulfide and nitrite removal from industrial wastewaters under denitrifying conditions. Biotechnol. Bioprocess Eng. 17, 661–668. doi: 10.1007/s12257-011-0677-3

[ref37] Du R. Cao S. Li B. Niu M. Wang S. Peng Y. (2017). Performance and microbial community analysis of a novel DEAMOX based on partial-denitrification and anammox treating ammonia and nitrate wastewaters. Water Res. 108, 46–56. doi: 10.1016/j.watres.2016.10.051, PMID: 27817892

[ref38] Du R. Cao S. Peng Y. Zhang H. Wang S. (2019). Combined partial Denitrification (PD)-Anammox: a method for high nitrate wastewater treatment. Environ. Int. 126, 707–716. doi: 10.1016/j.envint.2019.03.007, PMID: 30878866

[ref39] Duan H. Ye L. Lu X. Yuan Z. (2019). Overcoming nitrite oxidizing bacteria adaptation through alternating sludge treatment with free nitrous acid and free ammonia. Environ. Sci. Technol. 53, 1937–1946. doi: 10.1021/acs.est.8b06148, PMID: 30638367

[ref40] Dumestre A. Chone T. Portal J. Gerard M. Berthelin J. (1997). Cyanide degradation under alkaline conditions by a strain of Fusarium solani isolated from contaminated soils. Appl. Environ. Microbiol. 63, 2729–2734. doi: 10.1128/aem.63.7.2729-2734.1997, PMID: 16535647 PMC1389202

[ref41] Ezzi M. I. Lynch J. M. (2002). Cyanide catabolizing enzymes in Trichoderma spp. Enzym. Microb. Technol. 31, 1042–1047. doi: 10.1016/S0141-0229(02)00238-7

[ref42] Feleke Z. Araki K. Sakakibara Y. Watanabe T. Kuroda M. (1998). Selective reduction of nitrate to nitrogen gas in a biofilm-electrode reactor. Water Res. 32, 2728–2734. doi: 10.1016/S0043-1354(98)00018-9

[ref43] Fernández I. Dosta J. Fajardo C. Campos J. L. Mosquera-Corral A. Méndez R. (2012). Short- and long-term effects of ammonium and nitrite on the Anammox process. J. Environ. Manag. 95, S170–S174. doi: 10.1016/j.jenvman.2010.10.044, PMID: 21074312

[ref44] Fernández I. Vázquez-Padín J. R. Mosquera-Corral A. Campos J. L. Méndez R. (2008). Biofilm and granular systems to improve Anammox biomass retention. Biochem. Eng. J. 42, 308–313. doi: 10.1016/j.bej.2008.07.011

[ref45] Fux C. Velten S. Carozzi V. Solley D. Keller J. (2006). Efficient and stable nitritation and denitritation of ammonium-rich sludge dewatering liquor using an SBR with continuous loading. Water Res. 40, 2765–2775. doi: 10.1016/j.watres.2006.05.003, PMID: 16815527

[ref46] Ganigué R. Gabarró J. López H. Ruscalleda M. Balaguer M. D. Colprim J. (2010). Combining partial nitritation and heterotrophic denitritation for the treatment of landfill leachate previous to an anammox reactor. Water Sci. Technol. 61, 1949–1955. doi: 10.2166/wst.2010.968, PMID: 20388991

[ref47] Glass C. Silverstein J. (1998). Denitrification kinetics of high nitrate concentration water: pH effect on inhibition and nitrite accumulation. Water Res. 32, 831–839. doi: 10.1016/S0043-1354(97)00260-1

[ref48] Grady Jr C.L. Daigger G. T. Love N. G. Filipe C. D. (2011). Biological Wastewater Treatment, Boca Raton, FL: CRC Press.

[ref49] Gu J. Yang Q. Liu Y. (2018). Mainstream anammox in a novel A-2B process for energy-efficient municipal wastewater treatment with minimized sludge production. Water Res. 138, 1–6. doi: 10.1016/j.watres.2018.02.051, PMID: 29554513

[ref50] Gurbuz F. Ciftci H. Akcil A. (2009). Biodegradation of cyanide containing effluents by Scenedesmus obliquus. J. Hazard. Mater. 162, 74–79. doi: 10.1016/j.jhazmat.2008.05.008, PMID: 18554792

[ref51] Gurbuz F. Ciftci H. Akcil A. Karahan A. G. (2004). Microbial detoxification of cyanide solutions: a new biotechnological approach using algae. Hydrometallurgy 72, 167–176. doi: 10.1016/j.hydromet.2003.10.004

[ref52] Han M. Vlaeminck S. E. AL-Omari A. Wett B. Bott C. Murthy S.. (2016). Uncoupling the solids retention times of flocs and granules in mainstream deammonification: a screen as effective out-selection tool for nitrite oxidizing bacteria. Bioresour. Technol. 221, 195–204. doi: 10.1016/j.biortech.2016.08.115, PMID: 27639672

[ref53] Hellinga C. Schellen A. A. J. C. Mulder J. W. Van Loosdrecht M. C. M. Heijnen J. J. (1998). The sharon process: an innovative method for nitrogen removal from ammonium-rich waste water. Water Sci. Technol. 37, 135–142. doi: 10.2166/wst.1998.0350

[ref54] Hellinga C. Van Loosdrecht M. C. M. Heijnen J. J. (1999). Model based Design of a Novel Process for nitrogen removal from concentrated flows. Math. Comput. Model. Dyn. Syst. 5, 351–371. doi: 10.1076/mcmd.5.4.351.3678

[ref55] Huang P. Hogsett M. (2011). The robustness of ANAMMOX communities treating full-scale sidestream municipal anaerobic digester filtrate. Proc. Water Environ. Fed. 2011, 3147–3155. doi: 10.2175/193864711802721730

[ref56] Ingvorsen K. Højer-Pedersen B. Godtfredsen S. E. (1991). Novel cyanide-hydrolyzing enzyme from Alcaligenes xylosoxidans subsp. denitrificans. Appl. Environ. Microbiol. 57, 1783–1789. doi: 10.1128/aem.57.6.1783-1789.1991, PMID: 1872607 PMC183468

[ref57] Isabel San-Martín M. Mateos R. Carracedo B. Escapa A. Morán A. (2018). Pilot-scale bioelectrochemical system for simultaneous nitrogen and carbon removal in urban wastewater treatment plants. J. Biosci. Bioeng. 126, 758–763. doi: 10.1016/j.jbiosc.2018.06.008, PMID: 30042004

[ref58] Isaka K. Kimura Y. Matsuura M. Osaka T. Tsuneda S. (2017). First full-scale nitritation-anammox plant using gel entrapment technology for ammonia plant effluent. Biochem. Eng. J. 122, 115–122. doi: 10.1016/j.bej.2017.03.005

[ref59] Jaroszynski L. W. Cicek N. Sparling R. Oleszkiewicz J. A. (2012). Impact of free ammonia on anammox rates (anoxic ammonium oxidation) in a moving bed biofilm reactor. Chemosphere 88, 188–195. doi: 10.1016/j.chemosphere.2012.02.085, PMID: 22483855

[ref60] Jianping C. Tang D. Tang Z. Guo J. (2022). A novel sulfur-driven autotrophic denitrification coupled with bio-cathode system for bioelectricity generation and groundwater remediation. Water Sci. Technol. 86, 979–991 doi: 10.2166/wst.2022.216 36358041

[ref61] Jung S. Lee J. Park Y.-K. Kwon E. E. (2020). Bioelectrochemical systems for a circular bioeconomy. Bioresour. Technol. 300:122748. doi: 10.1016/j.biortech.2020.122748, PMID: 31937485

[ref62] Kamei T. Rujakom S. Nakano M. Maharjan A. K. Kazama F. (2022). Investigation of nitrite accumulation by hydrogenotrophic denitrification in a moving bed biofilm reactor for partial denitrification and anammox process. Water Sci. Technol. 85, 3396–3407. doi: 10.2166/wst.2022.187, PMID: 35771053

[ref63] Kapoor V. Elk M. Li X. Santo Domingo J. W. (2016). Inhibitory effect of cyanide on wastewater nitrification determined using SOUR and RNA-based gene-specific assays. Lett. Appl. Microbiol. 63, 155–161. doi: 10.1111/lam.12603, PMID: 27281632

[ref64] Kartal B. De Almeida N. M. Maalcke W. J. OP Den Camp H. J. Jetten M. S. M. Keltjens J. T. (2013). How to make a living from anaerobic ammonium oxidation. FEMS Microbiol. Rev. 37, 428–461. doi: 10.1111/1574-6976.12014, PMID: 23210799

[ref65] Katayama Y. Narahara Y. Inoue Y. Amano F. Kanagawa T. Kuraishi H. (1992). A thiocyanate hydrolase of Thiobacillus thioparus. A novel enzyme catalyzing the formation of carbonyl sulfide from thiocyanate. J. Biol. Chem. 267, 9170–9175. doi: 10.1016/S0021-9258(19)50404-5, PMID: 1577754

[ref66] Keisar I. Desitti C. Beliavski M. Epsztein R. Tarre S. Green M. (2021). A pressurized hydrogenotrophic denitrification reactor system for removal of nitrates at high concentrations. J. Water Process Eng. 42:102140. doi: 10.1016/j.jwpe.2021.102140

[ref67] Kim Y. M. Lee D. S. Park C. Park D. Park J. M. (2011). Effects of free cyanide on microbial communities and biological carbon and nitrogen removal performance in the industrial activated sludge process. Water Res. 45, 1267–1279. doi: 10.1016/j.watres.2010.10.003, PMID: 21047665

[ref68] Kjeldsen P. (1999). Behaviour of cyanides in soil and groundwater: a review. Water Air Soil Pollut. 115, 279–308. doi: 10.1023/A:1005145324157

[ref69] Klaus S. Baumler R. Rutherford B. Thesing G. Zhao H. Bott C. (2017). Startup of a partial Nitritation-Anammox MBBR and the implementation of pH-based aeration control. Water Environ. Res. 89, 500–508. doi: 10.2175/106143017X14902968254476, PMID: 28545601

[ref70] Lackner S. Gilbert E. M. Vlaeminck S. E. Joss A. Horn H. Van Loosdrecht M. C. M. (2014a). Full-scale partial nitritation/anammox experiences – An application survey. Water Res. 55, 292–303. doi: 10.1016/j.watres.2014.02.032, PMID: 24631878

[ref71] Lackner S. Thoma K. Gilbert E. M. Gander W. Schreff D. Horn H. (2014b). Start-up of a full-scale deammonification SBR-treating effluent from digested sludge dewatering. Water Sci. Technol. 71, 553–559. doi: 10.2166/wst.2014.421 25746647

[ref72] Ladipo-Obasa M. Forney N. Riffat R. Bott C. Debarbadillo C. De Clippeleir H. (2022). Partial denitrification–anammox (PdNA) application in mainstream IFAS configuration using raw fermentate as carbon source. Water Environ. Res. 94:e10711 doi: 10.1002/wer.10711 35388559

[ref73] Lai E. Senkpiel S. Solley D. Keller J. (2004). Nitrogen removal of high strength wastewater via nitritation/denitritation using a sequencing batch reactor. Water Sci. Technol. 50, 27–33. doi: 10.2166/wst.2004.0601, PMID: 15656292

[ref74] Landkamer L. L. Bucknam C. H. Figueroa L. A. (2015). Anaerobic nitrogen transformations in a gold-cyanide leach residue. Environ. Sci. Technol. Lett. 2, 357–361. doi: 10.1021/acs.estlett.5b00279

[ref75] Lawson C. E. Wu S. Bhattacharjee A. S. Hamilton J. J. Mcmahon K. D. Goel R.. (2017). Metabolic network analysis reveals microbial community interactions in anammox granules. Nat. Commun. 8:15416. doi: 10.1038/ncomms15416, PMID: 28561030 PMC5460018

[ref76] Le T. Peng B. Su C. Massoudieh A. Torrents A. Al-Omari A.. (2019a). Impact of carbon source and COD/N on the concurrent operation of partial denitrification and anammox. Water Environ. Res. 91, 185–197. doi: 10.1002/wer.1016, PMID: 30699248

[ref77] Le T. Peng B. Su C. Massoudieh A. Torrents A. Al-Omari A.. (2019b). Nitrate residual as a key parameter to efficiently control partial denitrification coupling with anammox. Water Environ. Res. 91, 1455–1465. doi: 10.1002/wer.1140, PMID: 31074914

[ref78] Leaković S. Mijatović I. Cerjan-Stefanović Š. Hodžić E. (2000). Nitrogen removal from fertilizer wastewater by ion exchange. Water Res. 34, 185–190. doi: 10.1016/S0043-1354(99)00122-0

[ref79] Lee J. W. Lee K. H. Park K. Y. Maeng S. K. (2010). Hydrogenotrophic denitrification in a packed bed reactor: effects of hydrogen-to-water flow rate ratio. Bioresour. Technol. 101, 3940–3946. doi: 10.1016/j.biortech.2010.01.022, PMID: 20144861

[ref80] Lemaire R. Marcelino M. Yuan Z. (2008). Achieving the nitrite pathway using aeration phase length control and step-feed in an SBR removing nutrients from abattoir wastewater. Biotechnol. Bioeng. 100, 1228–1236. doi: 10.1002/bit.21844, PMID: 18553405

[ref81] Li X. Klaus S. Bott C. He Z. (2018a). Status, challenges, and perspectives of mainstream Nitritation–Anammox for wastewater treatment. Water Environ. Res. 90, 634–649. doi: 10.2175/106143017X15131012153112, PMID: 30188280

[ref82] Li X. Klaus S. Bott C. He Z. (2018b). Status, challenges, and perspectives of mainstream Nitritation‐Anammox for wastewater treatment. Water Environ. Res. 90, 634–649. doi: 10.2175/106143017X15131012153112, PMID: 30188280

[ref83] Li X. Shi M. Zhang M. Li W. Xu P.-L. Wang Y.. (2022). Progresses and challenges in sulfur autotrophic denitrification-enhanced Anammox for low carbon and efficient nitrogen removal. Crit. Rev. Environ. Sci. Technol. 52, 1–16. doi: 10.1080/10643389.2022.2037967

[ref84] Liang D. He W. Li C. Wang F. Crittenden J. C. Feng Y. (2021). Remediation of nitrate contamination by membrane hydrogenotrophic denitrifying biofilm integrated in microbial electrolysis cell. Water Res. 188:116498. doi: 10.1016/j.watres.2020.116498, PMID: 33080455

[ref85] Ma B. Qian W. Yuan C. Yuan Z. Peng Y. (2017). Achieving mainstream nitrogen removal through coupling Anammox with Denitratation. Environ. Sci. Technol. 51, 8405–8413. doi: 10.1021/acs.est.7b01866, PMID: 28661139

[ref86] Ma B. Xu X. Wei Y. Ge C. Peng Y. (2020). Recent advances in controlling denitritation for achieving denitratation/anammox in mainstream wastewater treatment plants. Bioresour. Technol. 299:122697. doi: 10.1016/j.biortech.2019.122697, PMID: 31902637

[ref87] Magrí A. Ruscalleda M. Vilà A. Akaboci T. R. V. Balaguer M. D. Llenas J. M.. (2021). Scaling-up and long-term operation of a full-scale two-stage partial Nitritation-Anammox system treating landfill leachate. PRO 9:800. doi: 10.3390/pr9050800

[ref88] Mansell B. O. Schroeder E. D. (2002). Hydrogenotrophic denitrification in a microporous membrane bioreactor. Water Res. 36, 4683–4690. doi: 10.1016/S0043-1354(02)00197-5, PMID: 12448509

[ref89] Martienssen M. Schöps R. (1999). Population dynamics of denitrifying bacteria in a model biocommunity. Water Res. 33, 639–646. doi: 10.1016/S0043-1354(98)00222-X

[ref90] Meyers P. R. Rawlings D. E. Woods D. R. Lindsey G. G. (1993). Isolation and characterization of a cyanide dihydratase from Bacillus pumilus C1. J. Bacteriol. 175, 6105–6112. doi: 10.1128/jb.175.19.6105-6112.1993, PMID: 8407782 PMC206703

[ref91] Mohsenpour S. F. Hennige S. Willoughby N. Adeloye A. Gutierrez T. (2021). Integrating micro-algae into wastewater treatment: a review. Sci. Total Environ. 752:142168. doi: 10.1016/j.scitotenv.2020.142168, PMID: 33207512

[ref92] Moraes B. S. Souza T. S. O. Foresti E. (2012). Effect of sulfide concentration on autotrophic denitrification from nitrate and nitrite in vertical fixed-bed reactors. Process Biochem. 47, 1395–1401. doi: 10.1016/j.procbio.2012.05.008

[ref93] Mousazadeh M. Niaragh E. K. Usman M. Khan S. U. Sandoval M. A. Al-Qodah Z.. (2021). A critical review of state-of-the-art electrocoagulation technique applied to COD-rich industrial wastewaters. Environ. Sci. Pollut. Res. 28, 43143–43172. doi: 10.1007/s11356-021-14631-w, PMID: 34164789

[ref94] Mulder A. Versprille A. I. Van Braak D. (2012). Sustainable nitrogen removal by denitrifying anammox applied for anaerobic pre-treated potato wastewater. Water Sci. Technol. 66, 2630–2637. doi: 10.2166/wst.2012.466, PMID: 23109579

[ref95] Mulder J. W. Van Loosdrecht M. C. M. Hellinga C. Van Kempen R. (2001). Full-scale application of the SHARON process for treatment of rejection water of digested sludge dewatering. Water Sci. Technol. 43, 127–134. doi: 10.2166/wst.2001.0675, PMID: 11443954

[ref96] Nguyen V. K. Hong S. Park Y. Jo K. Lee T. (2015). Autotrophic denitrification performance and bacterial community at biocathodes of bioelectrochemical systems with either abiotic or biotic anodes. J. Biosci. Bioeng. 119, 180–187. doi: 10.1016/j.jbiosc.2014.06.016 25073684

[ref97] Noutsopoulos C. Mamais D. Statiris E. Lerias E. Malamis S. Andreadakis A. (2018). Reject water characterization and treatment through short-cut nitrification/denitrification: assessing the effect of temperature and type of substrate. J. Chem. Technol. Biotechnol. 93, 3638–3647. doi: 10.1002/jctb.5745

[ref98] Oshiki M. Masuda Y. Yamaguchi T. Araki N. (2018). Synergistic inhibition of anaerobic ammonium oxidation (anammox) activity by phenol and thiocyanate. Chemosphere 213, 498–506. doi: 10.1016/j.chemosphere.2018.09.055, PMID: 30245226

[ref99] Pan J. Ma J. Wu H. Ren Y. Fu B. He M.. (2018). Simultaneous removal of thiocyanate and nitrogen from wastewater by autotrophic denitritation process. Bioresour. Technol. 267, 30–37. doi: 10.1016/j.biortech.2018.07.014, PMID: 30007236

[ref100] Park H. Brotto A. C. Van Loosdrecht M. C. M. Chandran K. (2017a). Discovery and metagenomic analysis of an anammox bacterial enrichment related to Candidatus “Brocadia caroliniensis” in a full-scale glycerol-fed nitritation-denitritation separate centrate treatment process. Water Res. 111, 265–273. doi: 10.1016/j.watres.2017.01.011, PMID: 28088723

[ref101] Park H. Sundar S. Ma Y. Chandran K. (2015). Differentiation in the microbial ecology and activity of suspended and attached bacteria in a nitritation-anammox process. Biotechnol. Bioeng. 112, 272–279. doi: 10.1002/bit.25354, PMID: 25115980

[ref102] Park H. I. Kim J. S. Kim D. K. Choi Y.-J. Pak D. (2006). Nitrate-reducing bacterial community in a biofilm-electrode reactor. Enzym. Microb. Technol. 39, 453–458. doi: 10.1016/j.enzmictec.2005.11.028

[ref103] Park M.-R. Park H. Chandran K. (2017b). Molecular and kinetic characterization of planktonic Nitrospira spp. selectively enriched from activated sludge. Environ. Sci. Technol. 51, 2720–2728. doi: 10.1021/acs.est.6b05184, PMID: 28124895

[ref104] Pu J. Feng C. Liu Y. Li R. Kong Z. Chen N.. (2014). Pyrite-based autotrophic denitrification for remediation of nitrate contaminated groundwater. Bioresour. Technol. 173, 117–123. doi: 10.1016/j.biortech.2014.09.092, PMID: 25299487

[ref105] Qian J. Zhou J. Zhang Z. Liu R. Wang Q. (2016). Biological nitrogen removal through nitritation coupled with thiosulfate-driven denitritation. Sci. Rep. 6:27502. doi: 10.1038/srep27502, PMID: 27272192 PMC4897740

[ref106] Qin J.-J. Oo M. H. Tao G. Kekre K. A. (2007). Feasibility study on petrochemical wastewater treatment and reuse using submerged MBR. J. Membr. Sci. 293, 161–166. doi: 10.1016/j.memsci.2007.02.012

[ref107] Regmi P. Miller M. W. Holgate B. Bunce R. Park H. Chandran K.. (2014). Control of aeration, aerobic SRT and COD input for mainstream nitritation/denitritation. Water Res. 57, 162–171. doi: 10.1016/j.watres.2014.03.035, PMID: 24721663

[ref108] Reij M. W. De Bont J. A. M. Hartmans S. De Gooijer K. D. (1995). Membrane bioreactor with a porous hydrophobic membrane as a gas–liquid contactor for waste gas treatment. Biotechnol. Bioeng. 45, 107–115. doi: 10.1002/bit.260450203, PMID: 18623091

[ref109] Rezania B. Cicek N. Oleszkiewicz J. A. (2005). Kinetics of hydrogen-dependent denitrification under varying pH and temperature conditions. Biotechnol. Bioeng. 92, 900–906. doi: 10.1002/bit.20664, PMID: 16116656

[ref110] Sahinkaya E. Kilic A. Duygulu B. (2014). Pilot and full scale applications of sulfur-based autotrophic denitrification process for nitrate removal from activated sludge process effluent. Water Res. 60, 210–217. doi: 10.1016/j.watres.2014.04.052, PMID: 24862952

[ref111] Sander E. M. Virdis B. Freguia S. (2017). Bioelectrochemical nitrogen removal as a polishing mechanism for domestic wastewater treated effluents. Water Sci. Technol. 76, 3150–3159. doi: 10.2166/wst.2017.462, PMID: 29210701

[ref112] Sijbesma W. F. H. Almeida J. S. Reis M. A. M. Santos H. (1996). Uncoupling effect of nitrite during denitrification by *Pseudomonas fluorescens*: an in vivo 31P-NMR study. Biotechnol. Bioeng. 52, 176–182. doi: 10.1002/(SICI)1097-0290(19961005)52:1<176::AID-BIT18>3.0.CO;2-M, PMID: 18629864

[ref113] Song Y. Li Y. He X. Zhang H. Zhou M. Liu G. (2021). Recycling of residual valuable metals in cyanide-leached gold wastewater using the N263-TBP system. J. Environ. Chem. Eng. 9:106774. doi: 10.1016/j.jece.2021.106774

[ref114] Statiris E. Hadjimitsis E. Noutsopoulos C. Malamis S. (2021). Thiosulphate driven autotrophic denitrification via nitrite using synthetic wastewater. J. Chem. Technol. Biotechnol. 96, 1675–1681. doi: 10.1002/jctb.6692

[ref115] Suarez C. Persson F. Hermansson M. (2015). Predation of nitritation–anammox biofilms used for nitrogen removal from wastewater. FEMS Microbiol. Ecol. 91:fix124. doi: 10.1093/femsec/fiv124, PMID: 26472578

[ref116] Suh Y.-J. Park J. M. Yang J.-W. (1994). Biodegradation of cyanide compounds by Pseudomonas fluorescens immobilized on zeolite. Enzym. Microb. Technol. 16, 529–533. doi: 10.1016/0141-0229(94)90025-6

[ref117] Syron E. Casey E. (2008). Membrane-aerated biofilms for high rate biotreatment: performance appraisal, engineering principles, scale-up, and development requirements. Environ. Sci. Technol. 42, 1833–1844. doi: 10.1021/es0719428, PMID: 18409602

[ref118] Szekeres S. Kiss I. Kalman M. Soares M. I. M. (2002). Microbial population in a hydrogen-dependent denitrification reactor. Water Res. 36, 4088–4094. doi: 10.1016/S0043-1354(02)00130-6, PMID: 12405417

[ref119] Torrentó C. Cama J. Urmeneta J. Otero N. Soler A. (2010). Denitrification of groundwater with pyrite and Thiobacillus denitrificans. Chem. Geol. 278, 80–91. doi: 10.1016/j.chemgeo.2010.09.003

[ref120] Trapp S. Larsen M. Pirandello A. Danquah-Boakye J. (2003). Feasibility of cyanide elimination using plants. Eur. J. Mineral Process. Environ. Prot. 3, 128–137.

[ref121] Van Der Star W. R. Miclea A. I. Van Dongen U. G. Muyzer G. Picioreanu C. Van Loosdrecht M. C. (2008). The membrane bioreactor: a novel tool to grow anammox bacteria as free cells. Biotechnol. Bioeng. 101, 286–294. doi: 10.1002/bit.21891, PMID: 18421799

[ref122] Van Der Star W. R. L. Abma W. R. Blommers D. Mulder J.-W. Tokutomi T. Strous M.. (2007). Startup of reactors for anoxic ammonium oxidation: experiences from the first full-scale anammox reactor in Rotterdam. Water Res. 41, 4149–4163. doi: 10.1016/j.watres.2007.03.044, PMID: 17583763

[ref123] Van Kempen R. Mulder J. W. Uijterlinde C. A. Loosdrecht M. C. M. (2001). Overview: full scale experience of the SHARON® process for treatment of rejection water of digested sludge dewatering. Water Sci. Technol. 44, 145–152. doi: 10.2166/wst.2001.0035, PMID: 11496665

[ref124] Vilar A. Eiroa M. Kennes C. Veiga M. C. (2010). The SHARON process in the treatment of landfill leachate. Water Sci. Technol. 61, 47–52. doi: 10.2166/wst.2010.786, PMID: 20057090

[ref125] Vu H. P. Nguyen L. N. Wang Q. Ngo H. H. Liu Q. Zhang X.. (2022). Hydrogen sulphide management in anaerobic digestion: a critical review on input control, process regulation, and post-treatment. Bioresour. Technol. 346:126634. doi: 10.1016/j.biortech.2021.126634, PMID: 34971773

[ref126] Wang J. Gu J.-D. (2013). Dominance of Candidatus Scalindua species in anammox community revealed in soils with different duration of rice paddy cultivation in Northeast China. Appl. Microbiol. Biotechnol. 97, 1785–1798. doi: 10.1007/s00253-012-4036-x, PMID: 22526793 PMC3562551

[ref127] Wang Z. Xu X. Gong Z. Yang F. (2012). Removal of COD, phenols and ammonium from Lurgi coal gasification wastewater using A2O-MBR system. J. Hazard. Mater. 235-236, 78–84. doi: 10.1016/j.jhazmat.2012.07.012, PMID: 22902132

[ref128] Watanabe A. Yano K. Ikebukuro K. Karube I. (1998). Cyanide hydrolysis in a cyanide-degrading bacterium, *Pseudomonas stutzeri* AK61, by cyanidase. Microbiology 144, 1677–1682. doi: 10.1099/00221287-144-6-1677 9639937

[ref129] Wei Q. Kawagoshi Y. Huang X. Hong N. Van Duc L. Yamashita Y.. (2016). Nitrogen removal properties in a continuous marine anammox bacteria reactor under rapid and extensive salinity changes. Chemosphere 148, 444–451. doi: 10.1016/j.chemosphere.2016.01.041, PMID: 26845464

[ref130] Wett B. Omari A. Podmirseg S. Han M. Akintayo O. Gómez Brandón M.. (2013). Going for mainstream deammonification from bench to full scale for maximized resource efficiency. Water Sci. Technol. 68, 283–289. doi: 10.2166/wst.2013.150, PMID: 23863418

[ref131] Wett B. Podmirseg S. M. Gomez-Brandon M. Hell M. Nyhuis G. Bott C.. (2015). Expanding DEMON Sidestream Deammonification technology towards mainstream application. Water Environ. Res. 87, 2084–2089. doi: 10.2175/106143015X14362865227319, PMID: 26652120

[ref132] Wiemann M. Schenk H. Hegemann W. (1998). Anaerobic treatment of tannery wastewater with simultaneous sulphide elimination. Water Res. 32, 774–780. doi: 10.1016/S0043-1354(97)00309-6

[ref133] Wiesmann U. (1994). “Biological nitrogen removal from wastewater,” in Biotechnics/wastewater ed. A. Fiechter. (Berlin, Heidelberg: Springer Berlin Heidelberg).

[ref134] Wild S. R. Rudd T. Neller A. (1994). Fate and effects of cyanide during wastewater treatment processes. Sci. Total Environ. 156, 93–107. doi: 10.1016/0048-9697(94)90346-8

[ref135] Winkler M. K. H. Straka L. (2019). New directions in biological nitrogen removal and recovery from wastewater. Curr. Opin. Biotechnol. 57, 50–55. doi: 10.1016/j.copbio.2018.12.007, PMID: 30708205

[ref136] Woo Y. C. Lee J. J. Kim H.-S. (2022). Removal of nitrogen from municipal wastewater by denitrification using a sulfur-based carrier: a pilot-scale study. Chemosphere 296:133969. doi: 10.1016/j.chemosphere.2022.133969, PMID: 35181436

[ref137] Xie G.-J. Cai C. Hu S. Yuan Z. (2017). Complete nitrogen removal from synthetic anaerobic sludge digestion liquor through integrating Anammox and denitrifying anaerobic methane oxidation in a membrane biofilm reactor. Environ. Sci. Technol. 51, 819–827. doi: 10.1021/acs.est.6b04500, PMID: 27983816

[ref138] Yao H. Zhao X. Fan L. Jia F. Chen Y. Cai W.. (2022). Pilot-scale demonstration of one-stage partial nitritation/anammox process to treat wastewater from a coal to ethylene glycol (CtEG) plant. Environ. Res. 208:112540. doi: 10.1016/j.envres.2021.112540, PMID: 34915033

[ref139] Yu X. Nishimura F. Hidaka T. (2020). Anammox reactor exposure to thiocyanate: long-term performance and microbial community dynamics. Bioresour. Technol. 317:123960. doi: 10.1016/j.biortech.2020.123960, PMID: 32822893

[ref140] Zhang F. Peng Y. Wang Z. Jiang H. (2019). High-efficient nitrogen removal from mature landfill leachate and waste activated sludge (WAS) reduction via partial nitrification and integrated fermentation-denitritation process (PNIFD). Water Res. 160, 394–404. doi: 10.1016/j.watres.2019.05.032, PMID: 31163315

[ref141] Zhang J. Peng Y. Li X. Du R. (2022). Feasibility of partial-denitrification/anammox for pharmaceutical wastewater treatment in a hybrid biofilm reactor. Water Res. 208:117856. doi: 10.1016/j.watres.2021.117856, PMID: 34826739

[ref142] Zhang L. Narita Y. Gao L. Ali M. Oshiki M. Okabe S. (2017). Maximum specific growth rate of anammox bacteria revisited. Water Res. 116, 296–303. doi: 10.1016/j.watres.2017.03.027, PMID: 28347953

