# Supplementary material for: Biological nitrogen removal from low carbon wastewater
Source: Front Microbiol. 2022 Nov 16;13:968812. doi: 10.3389/fmicb.2022.968812 (PMC9709150; doi:10.3389/fmicb.2022.968812)
Supplement: Supplementary file 1 [file Data_Sheet_1.PDF]

Biological Nitrogen Removal from Low Carbon Wastewater

Kiprotich Kosgey*, Phumza Vuyokazi Zungu, Faizal Bux and Sheena Kumari**

Institute for Water and Wastewater Technology, Durban University of Technology, Durban, South Africa

Durban University of Technology, Institute for Water and Wastewater Technology, Durban, South Africa

**Corresponding author: sheenak1@dut.ac.za

*kiproticharapkosgey@gmail.com

Table S 1. Readiness of biological processes for pilot- and full-scale application

Process	Capacities of full-scale systems	Capacities of pilot-scale systems	Minimum C/N for complete nitrogen removal	Challenges	Benefits	References
Nitritation-denitritation	1800 m^3	250 L	1.71	✓ NOB growth requires regulation<br>✓ Requires COD supplementation	✓ High NRRs and NREs<br>✓ AOB are sensitive compared to AMX	(Ganigué et al., 2010, Mulder et al., 2001)
Partial nitritation-anammox	256 – 550 m^3	1.67-4 m^3	0.3	✓ NOB growth requires regulation<br>✓ Slow growth of anammox bacteria (AMX)<br>✓ Sensitivity of AMX to operating conditions	✓ Effluent limits can be achieved without COD addition<br>✓ High NRRs and NREs	(Dimitrova et al., 2020, Lackner et al., 2014, Lotti et al., 2015, Yokota et al., 2021, Wu et al., 2021)

Denitrifying ammonium oxidation (DEAMOX)	No records of application	360 L	0.57	✓ Requires COD supplementation<br>✓ Sensitivity of AMX to operating conditions	✓ No need to control NOB growth<br>✓ High NRRs and NREs	(Le et al., 2019)
Bioelectrochemical systems	No records of application	150 L	-	✓ Low NRRs<br>✓ Complex systems<br>✓ Affected by low conductivities of wastewater	✓ Electrical energy could be used instead of organic carbon	(Isabel San-Martín et al., 2018)
Autotrophic denitrification	10 m^3	3X25 L	2.86 (NO_3^- reduction)<br>1.71 (NO_2^- reduction)	✓ Metals, Sulphur and their ions/compounds can generate harmful chemicals<br>✓ Hydrogenotrophic denitrification requires complex systems<br>✓ Requires C/N≥2.86 for complete NO_3^- removal	✓ Moderate NRRs<br>✓ Most of the electron donors are available in different wastewaters	(Sahinkaya et al., 2014, Di Capua et al., 2019)

Additional references

DI CAPUA, F., PIROZZI, F., LENS, P. N. L. & ESPOSITO, G. 2019. Electron donors for autotrophic denitrification. *Chemical Engineering Journal,* 362**,** 922-937.

DIMITROVA, I., DABROWSKA, A. & EKSTRÖM, S. 2020. Start-up of a full-scale partial nitritation-anammox MBBR without inoculum at Klagshamn WWTP. *Water Science and Technology,* 81**,** 2033-2042.

GANIGUÉ, R., GABARRÓ, J., LÓPEZ, H., RUSCALLEDA, M., BALAGUER, M. D. & COLPRIM, J. 2010. Combining partial nitritation and heterotrophic denitritation for the treatment of landfill leachate previous to an anammox reactor. *Water Science and Technology,* 61**,** 1949-1955.

ISABEL SAN-MARTÍN, M., MATEOS, R., CARRACEDO, B., ESCAPA, A. & MORÁN, A. 2018. Pilot-scale bioelectrochemical system for simultaneous nitrogen and carbon removal in urban wastewater treatment plants. *Journal of Bioscience and Bioengineering,* 126**,** 758-763.

LACKNER, S., GILBERT, E. M., VLAEMINCK, S. E., JOSS, A., HORN, H. & VAN LOOSDRECHT, M. C. M. 2014. Full-scale partial nitritation/anammox experiences – An application survey. *Water Research,* 55**,** 292-303.

LE, T., PENG, B., SU, C., MASSOUDIEH, A., TORRENTS, A., AL-OMARI, A., MURTHY, S., WETT, B., CHANDRAN, K., DEBARBADILLO, C., BOTT, C. & DE CLIPPELEIR, H. 2019. Nitrate residual as a key parameter to efficiently control partial denitrification coupling with anammox. *Water Environment Research,* 91**,** 1455-1465.

LOTTI, T., KLEEREBEZEM, R., HU, Z., KARTAL, B., DE KREUK, M. K., VAN ERP TAALMAN KIP, C., KRUIT, J., HENDRICKX, T. L. & VAN LOOSDRECHT, M. C. 2015. Pilot-scale evaluation of anammox-based mainstream nitrogen removal from municipal wastewater. *Environ Technol,* 36**,** 1167-77.

MULDER, J. W., VAN LOOSDRECHT, M. C. M., HELLINGA, C. & VAN KEMPEN, R. 2001. Full-scale application of the SHARON process for treatment of rejection water of digested sludge dewatering. *Water Science and Technology,* 43**,** 127-134.

SAHINKAYA, E., KILIC, A. & DUYGULU, B. 2014. Pilot and full scale applications of sulfur-based autotrophic denitrification process for nitrate removal from activated sludge process effluent. *Water Research,* 60**,** 210-217.

WU, J., KONG, Z., LUO, Z., QIN, Y., RONG, C., WANG, T., HANAOKA, T., SAKEMI, S., ITO, M., KOBAYASHI, S., KOBAYASHI, M., XU, K.-Q., KOBAYASHI, T., KUBOTA, K. & LI, Y.-Y. 2021. A successful start-up of an anaerobic membrane bioreactor (AnMBR) coupled mainstream partial nitritation-anammox (PN/A) system: A pilot-scale study on in-situ NOB elimination, AnAOB growth kinetics, and mainstream treatment performance. *Water Research,* 207**,** 117783.

YOKOTA, N., MINESHIMA, R., WATANABE, Y., TOKUTOMI, T., KIYOKAWA, T., NISHIYAMA, T., FUJII, T. & FURUKAWA, K. 2021. Startup of pilot-scale single-stage nitrogen removal using anammox and partial nitritation (SNAP) reactor for waste brine treatment using marine anammox bacteria. *Journal of Bioscience and Bioengineering,* 132**,** 505-512.
